# Supplementary material for: Molecular Hybridization Strategy on the Design, Synthesis, and Structural Characterization of Ferrocene-N-acyl Hydrazones as Immunomodulatory Agents
Source: Molecules. 2022 Nov 30;27(23):8343. doi: 10.3390/molecules27238343 (PMC9737981; doi:10.3390/molecules27238343)
Supplement: Supplementary file 1 [file molecules-27-08343-s001.zip › molecules-1964833-supplementary.pdf]

Electronic Supplementary Information

Molecular hybridization strategy on the design, synthesis, and structural characterization of ferrocene-*N*-acyl hydrazones as immunomodulatory agents

Laís Peres Silva[1], Ivanilson Pimenta Santos[2], Dahara Keyse Carvalho Silva[2], Bruna Padilha Zurita Claro dos Reis[2], Cássio Santana Meira[1,2,3], Marcos Venícius Batista de Souza Castro[4], José Maurício dos Santos Filho[4], João Honorato de Araujo-Neto[5], Javier Alcides Ellena[5], Rafael Gomes da Silveira[5,6] and Milena Botelho Pereira Soares[2,3*]

[1] Departamento de Ciências da Vida, Universidade do Estado da Bahia (UNEB – State University of Bahia), Salvador 41150-000, BA, Brazil.
[2] Instituto Gonçalo Moniz, Fundação Oswaldo Cruz Foundation (IGM-FIOCRUZ/BA), 40296-710 Salvador, Bahia, Brazil.
[3] Instituto de Inovação em Sistemas Avançados de Saúde (CIMATEC ISI SAS – University Center SENAI/CIMATEC), Salvador 41650-010, BA, Brazil.
[4] Laboratory of Design and Synthesis Applied to Medicinal Chemistry-**SintMed**®, Centro de Tecnologia e Geociências, Universidade Federal de Pernambuco, 50740-521, Recife, PE, Brazil.
[5] Laboratory Multiuser Laboratory of Structural Crystallography, Physics Institute of São Carlos, University of São Paulo, 13566-590, São Carlos, SP, Brazil.
[6] Departamento de Química, Instituto Federal Goiano, Campus Ceres, 76300-000, Ceres, GO, Brazil.

Table of contents

Characterization of the ferrocenyl-*N*-acyl hydrazones **SintMed(141-156)** S2-S9
Spectroscopic data: NMR and IR spectra of Fc-NAH **SintMed(141-156)** S10-S44
X-ray crystallographic data of **SintMed149** S45-S47

Characterization of the ferrocenyl-*N*-acyl hydrazones **SintMed(141-156)**

(*E*)-*N'*-(Ferrocenylmethylidene)benzohydrazide **SintMed141**

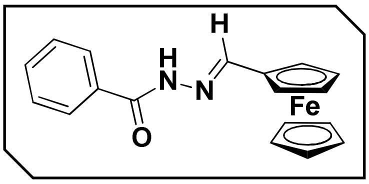

R_f 0.50 (AcOEt/Hexanes 1:1), red powder, yield 93%, mp 178.8-180.5 ºC (from dioxane/H_2O 1:1); IR (KBr, ν_{max} cm^{-1}): 3441, 3226 (CONH), 3063 (Ar CH), 1646 (C=O), 1607 (C=C), 1557 (C=N); ^{1}H NMR (400 MHz; DMSO-d_6, δH ppm): 11.5 (s, 1H, CONH), 8.29 (s, 1H, N=CH), 7.89 (d, 2H, ^3J = 6.9 Hz, Ar H-2,6), 7.57 (d, 1H, ^3J = 6.8 Hz, Ar H-4), 7.52 (d, 2H, ^3J = 6.8 Hz, Ar H-3,5), 4.66 (s, 2H, N=CH-Cp H-2,5), 4.46 (s, 2H, N=CH-Cp H-3,4), 4.24 (s, 5H, Cp-H);); ^{13}C NMR (100 MHz; DMSO-d_6, δH ppm): 162.3 (1C, C=O), 149.0 (1C, N=CH), 133.6 (1C, Ar C-1), 131.3 (1C, Ar C-4), 128.3 (2C, Ar C-3,5), 127.4 (2C, Ar C-2,6), 78.8 (1C, N=CH-Cp C-1), 70.1 (2C, N=CH-Cp C-3,4), 68.9 (5C, Cp), 67.5 (2C, N=CH-Cp C-2,5); Anal Calcd for $C_{18}H_{16}FeN_2O$: C, 65.08; H, 4.86; N, 8.43; Found: C, 65.01; H, 4.80; N, 8.51.

(*E*)-*N'*-(Ferrocenylmethylidene)-2-chlorobenzohydrazide **SintMed142**

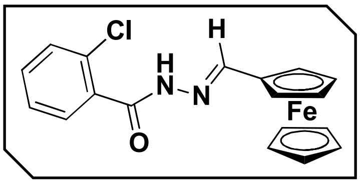

R_f 0.56 (AcOEt/Hexanes 1:1), red powder, yield 91%, mp 156.8-158.1 °C (from from dioxane/H_2O 1:1); IR (KBr, ν_{max} cm^{-1}): 3181 (CONH), 2993 (Aliphatic CH), 1641 (C=O), 1598 (C=C), 1547 (C=N); ^{1}H NMR (400 MHz; DMSO-d_6, δH ppm, ≈1.8:1 Rotamers mixture): 11.65 (s, CONH minor), 11.57 (s, 1H, CONH), 8.12 (s, 1H, N=CH), 7.88 (s, N=CH), 7.55-7.41 (m, 4H, Ar and minor), 4.66 (s, 2H, N=CH-Cp H-2,5), 4.46 (s, 2H, N=CH-Cp H-3,4), 4.34 (s, N=CH-Cp H-2,5 minor), 4.31 (s, N=CH-Cp H-3,4 minor), 4.23 (s, 5H, Cp-H), 4.15 (s, Cp-H minor); ^{13}C NMR (100 MHz; DMSO-d_6, δC ppm, ≈1.8:1 Rotamers mixture): 167.8 (CONH minor), 161.7 (1C, CONH), 149.1 (1C, N=CH), 144.5 (N=CH minor), 136.1 (Ar C-1 minor), 135.3 (1C, Ar C-1), 131.1, 130.3, 130.2, 129.8, 129.6, 129.2, 128.7, 128.5, 127.1, 126.7 (5C, Ar and minor), 78.8 (N=CH-Cp C-1 minor), 78.5 (1C, N=CH-Cp C-1), 70.2 (2C, N=CH-Cp C-3,4), 69.7 (N=CH-Cp C-3,4 minor), 68.9 (5C, Cp), 68.8 (5C, Cp minor), 67.6 (2C, N=CH-Cp C-2,5), 67.1 (2C, N=CH-Cp C-2,5 minor); Anal Calcd for $C_{18}H_{15}FeN_2ClO$: C, 58.97; H, 4.12; N, 7.64; Found: C, 58.86; H, 4.18; N, 7.54.

(*E*)-*N'*-(Ferrocenylmethylidene)furan-2-ylcarbohydrazide **SintMed143**

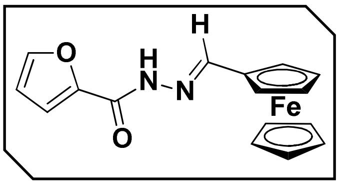

R_f 0.46 (AcOEt/Hexanes 1:1), dark red powder, yield 91%, mp 220.8-222.5 °C (from from dioxane/H_2O 1:1); IR (KBr, ν_{max} cm^{-1}): 3215 (CONH), 2925 (Aliphatic CH), 1651 (C=O), 1606 (C=C); ^1H NMR (300 MHz; DMSO-d_6, δH ppm): 11.5 (s, 1H, CONH), 8.28 (s, 1H, N=CH); 7.92 (s, 1H, Furyl H-5), 7.25 (s, 1H, Furyl H-3), 6.68 (s, 1H, Furyl H-4), 4.64 (s, 2H, N=CH-Cp H-2,5), 4.44 (s, 2H, N=CH-Cp H-3,4), 4.22 (s, 5H, Cp-H); ^{13}C NMR (75 MHz; DMSO-d_6, δC ppm): 153.6 (1C, CONH), 149.1 (1C, N=CH), 146.8 (1C, Furyl C-2), 145.4 (1C, Furyl C-5), 114.3 (1C, Furyl C-3), 111.9 (1C, Furyl C-4), 78.7 (1C, N=CH-Cp C-1), 70.1 (2C, N=CH-Cp C-3,4), 68.9 (5C, Cp), 67.5 (2C, N=CH-Cp C-2,5); Anal Calcd for $C_{16}H_{14}FeN_2O_2$: C, 59.66; H, 4.38; N, 8.70; Found: C, 59.57; H, 4.45; N, 8.78.

(*E*)-*N'*-(Ferrocenylmethylidene)-4-cyanobenzohydrazide **SintMed144**

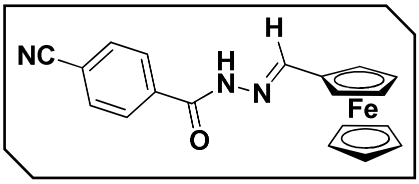

R_f 0.50 (AcOEt/Hexanes 1:1), red powder, yield 98%, mp 236.0-237.9 °C (from from dioxane/H_2O 1:1); IR (KBr, ν_{max} cm^{-1}): 3354, 3229 (CONH), 3090 (Ar CH), 2227 (C≡N), 1650 (C=O), 1611 (C=C), 1565 (C=N); ^1H NMR (300 MHz; DMSO-d_6, δH ppm): 11.8 (s, 1H, CONH), 8.31 (s, 1H, N=CH), 8.05 (br s, 2H, Ar), 8.02 (br s, 2H, Ar), 4.67 (s, 2H, N=CH-Cp H-2,5), 4.45 (s, 2H, N=CH-Cp H-3,4), 4.22 (s, 5H, Cp-H); ^{13}C NMR (75 MHz; DMSO-d_6, δC ppm): 160.9 (1C, CONH), 150.2 (1C, N=CH), 137.6 (1C, Ar C-1), 132.4 (2C, Ar), 128.3 (2C, Ar), 118.2 (1C, CN), 113.7 (1C, Ar C-4), 78.4 (1C, N=CH-Cp C-1), 70.3 (2C, N=CH-Cp C-3,4), 68.9 (5C, Cp), 67.6 (2C, N=CH-Cp C-2,5); Anal Calcd for $C_{19}H_{15}FeN_3O$: C, 63.89; H, 4.23; N, 11.76; Found: C, 63.99; H, 4.18; N, 11.71.

(*E*)-*N'*-(Ferrocenylmethylidene)quinolin-2-ylcarbohydrazide **SintMed145**

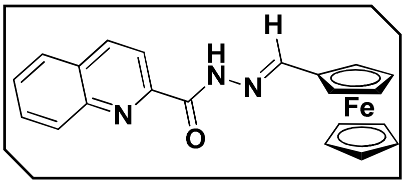

R_f 0.66 (AcOEt/Hexanes 1:1), red powder, yield 91%, mp 227.1-228.7 °C (from from dioxane/H_2O 1:1); IR (KBr, ν_{max} cm^{-1}): 3238 (CONH), 3050 (Ar CH), 1664 (C=O), 1600 (C=C), 1533 (C=N); ^1H NMR (400 MHz; DMSO-d_6, δH ppm): 11.9 (s, 1H, CONH), 8.60 (d, 1H, ^3J = 8.4 Hz, Quinoline H-4), 8.52 (s, 1H, N=CH),

8.21 (d, 1H, ^3J = 8.0 Hz, Quinoline H-3), 8.20 (d, 1H, ^3J = 8.0 Hz, Quinoline H-8), 8.11 (d, 1H, ^3J = 8.4 Hz, Quinoline H-5), 7.92 (t, 1H, ^3J = 7.2 Hz, Quinoline H-7), 7.75 (t, 1H, ^3J = 7.6 Hz, Quinoline H-6), 4.69 (s, 2H, N=CH-Cp H-2,5), 4.48 (s, 2H, N=CH-Cp H-3,4), 4.27 (s, 5H, Cp-H); ^{13}C NMR (100 MHz; DMSO-d_6, δC ppm): 159.7 (1C, C=O), 150.6 (1C, N=CH), 150.0 (1C, Quinoline C-2), 145.8 (1C, Quinoline C-8a), 137.9 (1C, Quinoline C-4), 130.5 (1C, Quinoline C-7), 129.1 (1C, Quinoline C-8), 128.8 (1C, Quinoline C-4a), 128.12 (1C, Quinoline C-5), 128.09 (1C, Quinoline C-6), 118.9 (1C, Quinoline C-3), 78.7 (1C, N=CH-Cp C-1), 70.2 (2C, N=CH-Cp C-3,4), 69.0 (5C, Cp), 67.6 (2C, N=CH-Cp C-2,5); Anal Calcd for $C_{21}H_{17}FeN_3O$: C, 65.82; H, 4.47; N, 10.96; Found: C, 65.75; H, 4.52; N, 11.03.

(*E*)-*N'*-(Ferrocenylmethylidene)-3,5-*tert*-butyl-4-hydroxybenzohydrazide **SintMed146**

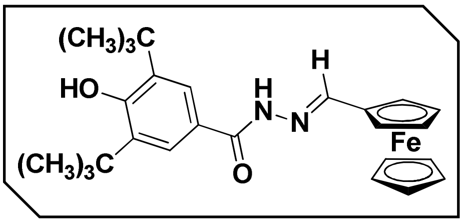

R_f 0.76 (AcOEt/Hexanes 1:1), red powder, yield 97%, mp 256.2-258.4 °C (dec, from from dioxane/H_2O 1:1); IR (KBr, ν_{max} cm^{-1}): 3627, 3610 (br OH), 3507, 3223 (CONH), 3082 (Ar CH), 2912 (Aliphatic CH), 1640 (C=O), 1607 (C=C), 1556 (C=N); ^{1}H NMR (400 MHz; DMSO-d_6, δH ppm): 11.3 (s, 1H, CONH), 8.29 (s, 1H, N=CH), 7.63 (s, 3H, OH and Ar H-2,6), 4.64 (s, 2H, N=CH-Cp H-2,5), 4.43 (s, 2H, N=CH-Cp H-3,4), 4.22 (s, 5H, Cp-H), 1.42 (s, 18H, 2x^{t}Bu); ^{13}C NMR (100 MHz; DMSO-d_6, δC ppm): 163.3 (1C, C=O), 156.9 (1C, Ar C-4), 148.2 (1C, N=CH), 138.3 (2C, Ar C-3,5), 124.8 (1C, Ar C-1), 124.2 (2C, Ar C-2,6), 79.2 (1C, N=CH-Cp C-1), 69.9 (2C, N=CH-Cp C-3,4), 68.9 (5C, Cp), 67.4 (2C, N=CH-Cp C-2,5), 34.5 (2C, ***C***^{t}Bu), 30.1 (6C, ^{t}Bu); Anal Calcd for $C_{26}H_{32}FeN_2O_2$: C, 67.83; H, 7.01; N, 6.08; Found: C, 67.89; H, 6.94; N, 6.13.

(*E*)-*N'*-(Ferrocenylmethylidene)-4-(trifluoromethyl)benzohydrazide **SintMed147**

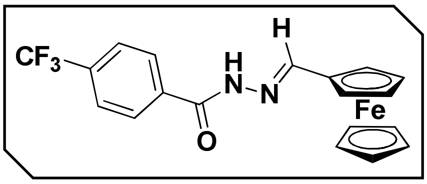

R_f 0.74 (AcOEt/Hexanes 1:1), red powder, yield 99%, mp 220.9-222.5 °C (from dioxane/H_2O 1:1); IR (KBr, ν_{max} cm^{-1}): 3355, 3201 (CONH), 3083, 3053 (Ar CH), 1657 (C=O), 1608 (C=C), 1564 (C=N); ^{1}H NMR (400 MHz; DMSO-d_6, δH ppm): 11.7 (s, 1H, CONH), 8.32 (s, 1H, N=CH), 8.10 (d, 2H, ^3J = 5.2 Hz, Ar H-2,6), 7.90 (d, 2H, ^3J = 6.0 Hz, Ar H-3,5), 4.68 (s, 2H, N=CH-Cp H-2,5), 4.47 (s, 2H, N=CH-Cp H-3,4), 4.24 (s, 5H, Cp-H); ^{13}C NMR (100 MHz; DMSO-d_6, δC ppm): 161.1 (1C, C=O), 150.0 (1C,

N=CH), 137.4 (1C, Ar C-1), 131.3 (q, 1C, $^{2}J_{FC}$ = 31 Hz, Ar C-4), 128.3 (2C, Ar C-2,6), 125.3 (2C, Ar C-3,5), 126.1 (q, 1C, $^{1}J_{FC}$ = 200 Hz, CF_3), 78.5 (1C, N=CH-Cp C-1), 70.2 (2C, N=CH-Cp C-3,4), 68.9 (5C, Cp), 67.6 (2C, N=CH-Cp C-2,5); ^{19}F NMR (376 MHz; DMSO-d_6, δF ppm): -61.2 (s, 1F, CF_3); Anal Calcd for $C_{19}H_{15}FeN_2F_3O$: C, 57.03; H, 3.78; N, 7.00; Found: C, 56.99; H, 3.83; N, 7.03.

(*E*)-*N'*-(Ferrocenylmethylidene)-2-methylbenzohydrazide **SintMed148**

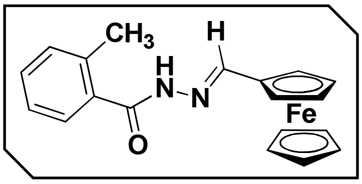

R_f 0.55 (AcOEt/Hexanes 1:1), red powder, yield 93%, mp 207.9-209.8 °C (from dioxane/H_2O 1:1); IR (KBr, ν_{max} cm^{-1}): 3174 (CONH), 3056 (Ar CH), 2991 (Aliphatic CH), 1643 (C=O), 1600 (C=C), 1559 (C=N); ^{1}H NMR (400 MHz; DMSO-d_6, δH ppm, ≈7:3 Rotamers mixture):11.4 (s, 1H, CONH and minor), 8.15 (s, 1H, N=CH), 7.89 (s, N=CH minor), 7.43-7.29 (m, 4H, Ar H-3,4,5,6 and minor), 4.64 (s, 2H, N=CH-Cp H-2,5), 4.44 (s, 2H, N=CH-Cp H-3,4), 4.36 (s, N=CH-Cp H-2,5 minor), 4.31 (s, N=CH-Cp H-3,4 minor), 4.23 (s, 5H, Cp-H), 4.17 (s, Cp-H minor), 2.38 (s, 3H, CH_3), 2.30 (s, CH_3 minor); ^{13}C NMR (100 MHz; DMSO-d_6, δC ppm, ≈7:3 Rotamers mixture): 170.5 (CONH minor), 164.4 (1C, CONH), 148.4 (1C, N=CH), 144.0 (N=CH minor), 136.1, 135.6, 135.4, 134.4, 130.4, 129.6, 129.4, 128.7, 127.24, 127.17, 125.4, 124.9 (6C, Ar and minor), 78.9 (N=CH-Cp C-1 minor), 78.8 (1C, N=CH-Cp C-1), 70.0 (2C, N=CH-Cp C-3,4), 69.6 (N=CH-Cp C-3,4 minor), 68.8 (5C, Cp), 68.7 (Cp minor), 67.4 (2C, N=CH-Cp C-2,5), 67.0 (N=CH-Cp C-2,5 minor), 19.23 (CH_3 minor), 19.18 (1C, CH_3); Anal Calcd for $C_{19}H_{18}FeN_2O$: C, 65.92; H, 5.24; N, 8.09; Found: C, 65.99; H, 5.20; N, 8.15.

(*E*)-*N'*-(Ferrocenylmethylidene)-2-bromobenzohydrazide **SintMed149**

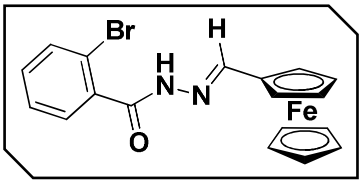

R_f 0.70 (AcOEt/Hexanes 1:1), red powder, yield 87%, mp 163.4-165.8 °C (dec, from dioxane/H_2O 1:1); IR (KBr, ν_{max} cm^{-1}): 3190 (CONH), 3055 (Ar CH), 1656 (C=O), 1600 (C=C), 1562 (C=N); ^{1}H NMR (400 MHz; DMSO-d_6, δH ppm, ≈2:1 Rotamers mixture): 11.6 (s, CONH minor), 11.5 (s, 1H, CONH), 8.11 (s, 1H, N=CH), 7.87 (s, N=CH minor), 7.71 (d, 1H, ^{3}J = 8.0 Hz, Ar H-6), 7.68 (d, ^{3}J = 8.0 Hz, Ar H-6 minor), 7.54-7.37 (m, 3H, Ar H-3,4,5 and minor), 4.66 (s, 2H, N=CH-Cp H-2,5), 4.46 (s, 2H, ^{3}J = 2.8 Hz, N=CH-Cp H-3,4), 4.34 (s, N=CH-Cp H-2,5 minor), 4.31 (s, N=CH-Cp H-3,4 minor), 4.24 (s,

5H, Cp-H), 4.16 (s, Cp-H minor); ^{13}C NMR (100 MHz; DMSO-d_6, δC ppm): 162.5 (1C, CONH), 149.0 (1C, N=CH), 144.4 (N=CH minor), 137.5, 132.7, 131.9, 131.2, 130.2, 129.2, 128.5, 127.6, 127.1 (5C, Ar C-1,3,4,5,6 and minor), 119.4 (1C, Ar C-2), 78.9 (N=CH-Cp C-1 minor), 78.6 (1C, N=CH-Cp C-1), 70.2 (2C, N=CH-Cp C-3,4), 69.7 (N=CH-Cp C-3,4 minor), 68.91 (5C, Cp), 68.86 (Cp minor), 67.6 (2C, N=CH-Cp C-2,5), 67.1 (N=CH-Cp C-2,5 minor); Anal Calcd for $C_{18}H_{15}FeN_2BrO$: C, 52.59; H, 3.68; N, 6.81; Found: C, 52.52; H, 3.71; N, 6.90.

(*E*)-*N*'-(Ferrocenylmethylidene)-2-phenoxybenzohydrazide **SintMed150**

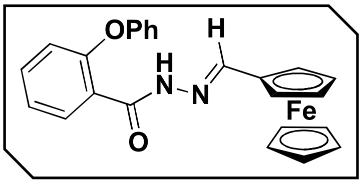

R$_f$ 0.65 (AcOEt/Hexanes 1:1), red powder, yield 83%, mp 177.9-179.5 ºC (dec, from dioxane/H_2O 1:1); IR (KBr, ν_{max} cm^{-1}): 3308 (CONH), 3060 (Ar CH), 1661 (C=O), 1602 (C=C), 1541 (C=N); ^{1}H NMR (400 MHz; DMSO-d_6, δH ppm, ≈7:3 Rotamers mixture): 11.45 (s, CONH minor), 11.38 (s, 1H, CONH), 8.14 (s, 1H, N=CH), 7.84 (s, N=CH minor), 7.67-6.98 (m, 9H, Ar and OPh and minor), 4.62 (s, 2H, N=CH-Cp H-2,5), 4.42 (s, 2H, N=CH-Cp H-3,4), 4.39 (s, N=CH-Cp H-2,5 minor), 4.32 (s, N=CH-Cp H-3,4 minor), 4.20 (s, 5H, Cp-H), 4.12 (s, Cp-H minor); ^{13}C NMR (100 MHz; DMSO-d_6, δC ppm, ≈7:3 Rotamers mixture): 167.9 (CONH minor), 161.1 (1C, CONH), 156.5, 153.0 (2C, Ar), 148.4 (1C, N=CH), 144.2 (N=CH minor), 131.7, 130.4, 130.2, 129.8, 129.6, 128.8, 128.6, 127.5, 123.6, 123.4, 123.1, 122.9, 120.1, 119.1, 118.4, 118.3, 118.0 (10C, Ar, OPh and minor), 78.9 (N=CH-Cp C-1 minor), 78.7 (1C, N=CH-Cp C-1), 70.1 (2C, N=CH-Cp C-3,4), 69.7 (N=CH-Cp C-3,4 minor), 68.8 (5C, Cp), 68.7 (Cp minor), 67.5 (2C, N=CH-Cp C-2,5), 67.1 (N=CH-Cp C-2,5 minor); Anal Calcd for $C_{24}H_{20}FeN_2O_2$: C, 67.94; H, 4.75; N, 6.60; Found: C, 67.87; H, 4.82; N, 6.65.

(*E*)-*N*'-(Ferrocenylmethylidene)-3,4,5-trihydroxybenzohydrazide **SintMed151**

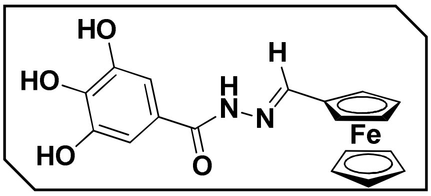

R$_f$ 0.10 (AcOEt), red powder, yield 96%, mp 305.3-306.0 °C (dec, from dioxane/H_2O 1:1); IR (KBr, ν_{max} cm^{-1}): 3552-3000 (br, OH), 3225 (CONH), 3084 (Ar CH), 1633 (C=O), 1608 (C=C), 1570 (C=N); ^{1}H NMR (300 MHz; DMSO-d_6, δH ppm): 11.2 (s, 1H, CONH), 9.13 (s, 2H, 3,5-OH), 8.77 (s, 1H, 4-OH), 8.23 (s, 1H, N=CH), 6.90 (s, 2H, Ar H-2,6), 4.62 (s, 2H, N=CH-Cp H-2,5), 4.42 (s, 2H, N=CH-Cp H-

3,4), 4.21 (s, 5H, Cp-H); ^{13}C NMR (75 MHz; DMSO-*d*$_6$, δC ppm): 162.5 (1C, C=O), 147.8 (1C, N=CH), 145.4 (2C, Ar C-3,5), 136.6 (1C, Ar C-4), 123.6 (1C, Ar C-1), 106.9 (2C, Ar C-2,6), 79.2 (1C, N=CH-Cp C-1), 69.9 (2C, N=CH-Cp C-3,4), 68.9 (5C, Cp), 67.4 (2C, N=CH-Cp C-2,5); Anal Calcd for $C_{18}H_{16}FeN_2O_4$: C, 56.87; H, 4.24; N, 7.37; Found: C, 56.82; H, 4.31; N, 7.32.

(*E*)-*N'*-(Ferrocenylmethylidene)-3,4,5-trimethoxybenzohydrazide **SintMed152**

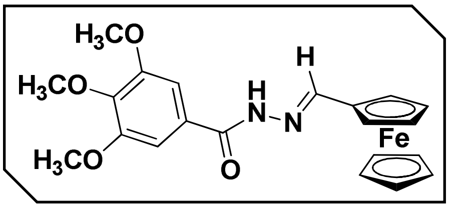

R_f 0.18 (AcOEt/Hexanes 1:1), red powder, yield 90%, mp 237.8-239.5 °C (dec, from dioxane/H_2O 1:1); IR (KBr, ν_{max} cm^{-1}): 3235 (CONH), 3083 (Ar CH), 2942 (Aliphatic CH), 1646 (C=O), 1608 (C=C), 1582 (C=N); ^{1}H NMR (400 MHz, DMSO-d$_6$, δH ppm): 11.4 (s, 1H, CONH), 8.32 (s, 1H, N=CH), 7.23 (s, 2H, Ar H-2,6), 4.66 (s, 2H, N=CH-Cp H-2,5), 4.45 (s, 2H, N=CH-Cp H-3,4), 4.23 (s, 5H, Cp-H), 3.86 (s, 6H, 3,5-OCH_3), 3.73 (s, 3H, 4-OCH_3); ^{13}C NMR (100 MHz, DMSO-d$_6$, δC ppm): 161.8 (1C, C=O), 152.6 (2C, Ar C-3,5), 149.1 (1C, N=CH), 140.2 (1C, Ar C-4), 128.7 (1C, Ar C-1), 105.0 (2C, Ar C-2,6), 78.8 (1C, N=CH-Cp C-1), 70.1 (2C, N=CH-Cp C-3,4), 68.9 (5C, Cp), 67.5 (2C, N=CH-Cp C-2,5), 60.0 (1C, 4-OCH_3), (2C, 3,5-OCH_3); Anal Calcd for $C_{21}H_{22}FeN_2O_4$: C, 59.73; H, 5.25; N, 6.63; Found: C, 59.65; H, 5.22; N, 6.70.

(*E*)-*N'*-(Ferrocenylmethylidene)-3,4,5-trimethoxybenzohydrazide **SintMed153**

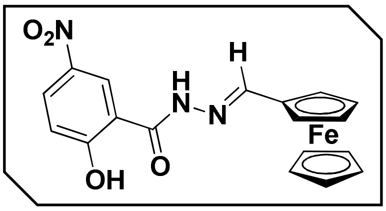

R_f 0.18 (AcOEt/Hexanes 1:1), yellow powder, yield 86%, mp 221.4-222.6 °C (dec, from dioxane/H_2O 1:1); IR (KBr, ν_{max} cm^{-1}): 3379, 3328, 3255 (CONH, OH), 3095 (Ar CH), 1630 (C=O), 1558 (C=N); ^{1}H NMR (400 MHz; DMSO-*d*$_6$, δH ppm, ≈2:1 Rotamers mixture): 13.6 (s, 1H, CONH), 9.23 (s, 1H, Ar H-6), 9.11 (s, Ar H-6 minor), 8.37 and 8.35 (2s, 1H, Ar H-4 major and minor), 8.12 (s, 1H, N=CH), 7.34 (s, 1H, Ar H-3), 4.50 (s, 2H, N=CH-Cp H-2,5), 4.38 (s, 2H, N=CH-Cp H-3,4), 4.16 (s, 5H, Cp-H); ^{13}C NMR (100 MHz; DMSO-*d*$_6$, δC ppm): 167.1 (1C, CONH), 149.0 (1C, N=CH), 136.1 (1C, Ar C-2), 134.4 (1C, Ar C-5), 128.1 (1C, Ar C-4), 127.4 (1C, Ar C-6), 126.3 (1C, Ar C-1), 120.9 (1C, Ar C-3), 78.7 (1C, N=CH-Cp C-1), 69.9 (2C, N=CH-Cp C-3,4), 68.8 (5C, Cp), 67.5 (2C, N=CH-Cp C-2,5); Anal Calcd for $C_{18}H_{15}FeN_3O_4$: C, 54.99; H, 3.85; N, 10.69; Found: C, 54.92; H, 3.84; N, 10.74.

(*E*)-*N'*-(Ferrocenylmethylidene)-3-chlorobenzohydrazide **SintMed154**

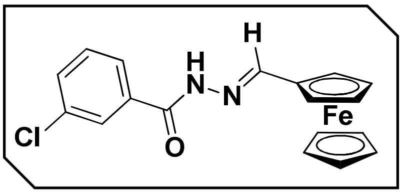

R_f 0.53 (AcOEt/Hexanes 3:7), red powder, yield 98%, mp 225.7-228.2 °C (dec, from dioxane/H_2O 1:1); IR (KBr, ν_{max} cm^{-1}): 3196 (CONH), 3063 (Ar CH), 1643 (C=O), 1608 (C=C), 1568 (C=N); ^1H NMR (400 MHz, DMSO-d$_6$, δH ppm): 11.6 (s, 1H, CONH), 8.29 (s, 1H, N=CH), 7.95 (s, 1H, Ar H-2), 7.86 (d, 1H, ^3J = 6.8 Hz, Ar H-6), 7.65 (d, 1H, ^3J = 7.6 Hz, Ar H-4), 7.55 (t, 1H, ^3J = 7.6 Hz, Ar H-5), 4.67 (s, 2H, N=CH-Cp H-2,5), 4.46 (s, 2H, N=CH-Cp H-3,4), 4.23 (s, 5H, Cp); ^{13}C NMR (100 MHz, DMSO-*d*$_6$, δC ppm): 160.8 (C=O), 149.7 (N=CH), 135.6 (1C, Ar C-3), 133.1 (1C, Ar C-1), 131.2 (1C, Ar C-4), 130.3 (1C, Ar C-5), 127.1 (1C, Ar C-2), 126.2 (1C, Ar C-6), 78.6 (1C, N=CH-Cp C-1), 70.2 (2C, N=CH-Cp C-3,4), 68.9 (5C, Cp), 67.5 (2C, N=CH-Cp C-2,5); Anal Calcd for $C_{18}H_{15}FeN_2ClO$: C, 58.97; H, 4.12; N, 7.64; Found: C, 59.04; H, 4.09; N, 7.58.

(*E*)-*N'*-(Ferrocenylmethylidene)-1-naphtylcarbohydrazide **SintMed155**

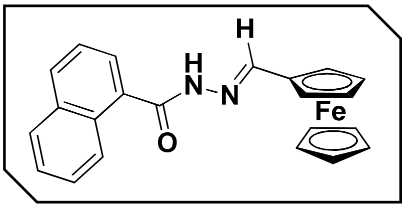

R_f 0.68 (AcOEt/Hexanes 1:1), red powder, yield 93%, mp 233.0-234.4 °C (dec, from from dioxane/H_2O 1:1); IR (KBr, ν_{max} cm^{-1}): 3185 (CONH), 3035 (Ar CH), 2977, 2845 (Aliphatic CH), 1636 (C=O), 1617 (C=C), 1564 (C=N); ^1H NMR (400 MHz; DMSO-*d*$_6$, δH ppm): 11.7 (s, 1H, CONH), 8.24-8.22 (m, 1H, Naphtyl), 8.20 (s, 1H, N=CH), 8.08 (d, 1H, ^3J = 8.0 Hz, Naphtyl), 8.03-8.00 (m, 1H, Naphtyl), 7.74 (d, 1H, ^3J = 7.2 Hz, Naphtyl), 7.61-7.58 (m, 3H, Naphtyl), 4.68 (s, 2H, N=CH-Cp H-2,5), 4.47 (s, 2H, N=CH-Cp H-3,4), 4.26 (s, 5H, Cp-H); ^{13}C NMR (100 MHz; DMSO-*d*$_6$, δC ppm): 163.9 (C=O), 148.9 (N=CH), 133.1, 133.0, 130.2, 130.0, 128.2, 126.8, 126.3, 125.6, 125.1, 124.9 (10C, Naphtyl), 78.8 (1C, N=CH-Cp C-1), 70.1 (2C, N=CH-Cp C-3,4), 68.9 (5C, Cp), 67.5 (2C, N=CH-Cp C-2,5); Anal Calcd for $C_{22}H_{18}FeN_2O$: C, 69.13; H, 4.75; N, 7.33; Found: C, 69.21; H, 4.73; N, 7.36.

(*E*)-*N'*-(Ferrocenylmethylidene)-2,4-dimethoxybenzohydrazide **SintMed156**

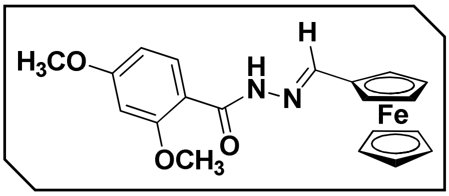

R_f 0.32 (AcOEt/Hexanes 1:1), red powder, yield 87%, mp 123.9-126.6 °C (dec, from dioxane/H_2O 1:1); IR

(KBr, ν_{max} cm^{-1}): 3223 (CONH), 3083 (Ar CH), 2836 (Aliphtic CH), 1641 (C=O), 1603 (C=C), 1561 (C=N); ^{1}H NMR (400 MHz; DMSO-d_6, δH ppm): 11.4 (s, 1H, CONH), 8.30 (s, 1H, N=CH), 7.55 (d, 1H, ^{3}J = 8.0 Hz, Ar H-5), 7.48 (s, 1H, Ar H-3), 7.07 (d, 1H, ^{3}J = 8.8 Hz, Ar H-6), 4.65 (s, 2H, N=CH-Cp H-2,5), 4.44 (s, 2H, N=CH-Cp H-3,4), 4.23 (s, 5H, Cp-H), 3.84 (s, 3H, OCH_3), 3.83 (s, 3H, OCH_3); ^{13}C NMR (100 MHz; DMSO-d_6, δC ppm): 161.8 (C=O), 151.4 (1C, Ar C-4), 148.5 (1C, Ar C-2), 148.2 (N=CH), 125.6 (Ar C-1), 120.7 (1C, Ar C-5), 110.9 (1C, Ar C-6), 110.7 (1C, Ar C-3), 79.0 (1C, N=CH-Cp C-1), 70.0 (2C, N=CH-Cp C-3,4), 68.9 (5C, Cp), 67.4 (2C, N=CH-Cp C-2,5), 55.5 (2C, 2xOCH_3); Anal Calcd for $C_{20}H_{20}FeN_2O_3$: C, 61.24; H, 5.14; N, 7.14; Found: C, 61.16; H, 5.15; N, 7.09.

NMR and IR spectra of Fc-NAH **SintMed(141-156)**

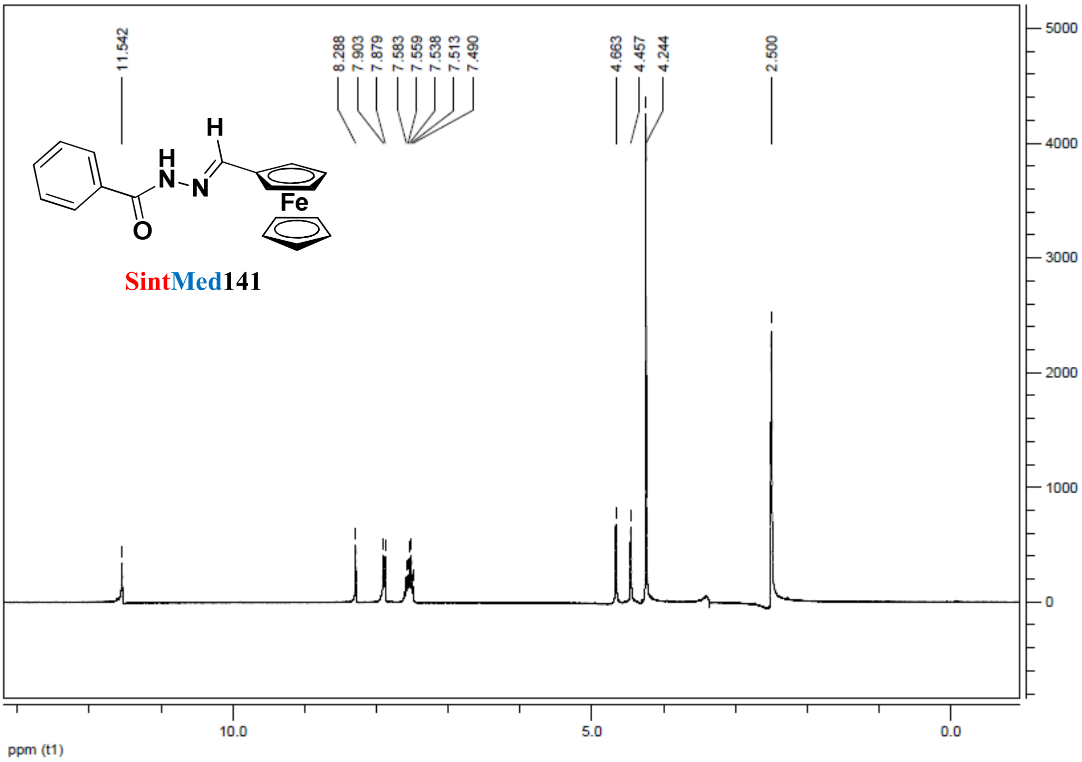

^{1}H NMR spectrum of SintMed141 (400 MHz, DMSO-*d*$_6$)

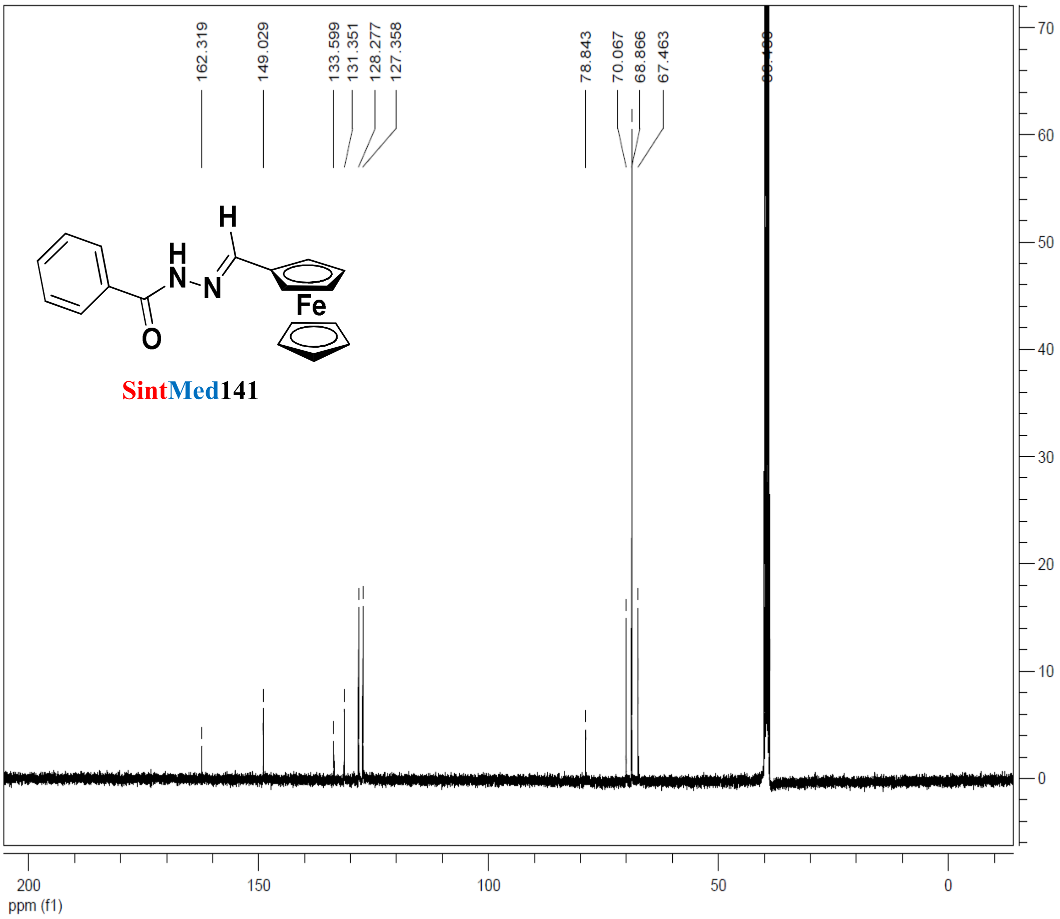

^{13}C NMR spectrum of SintMed141 (100 MHz, DMSO-*d*$_6$)

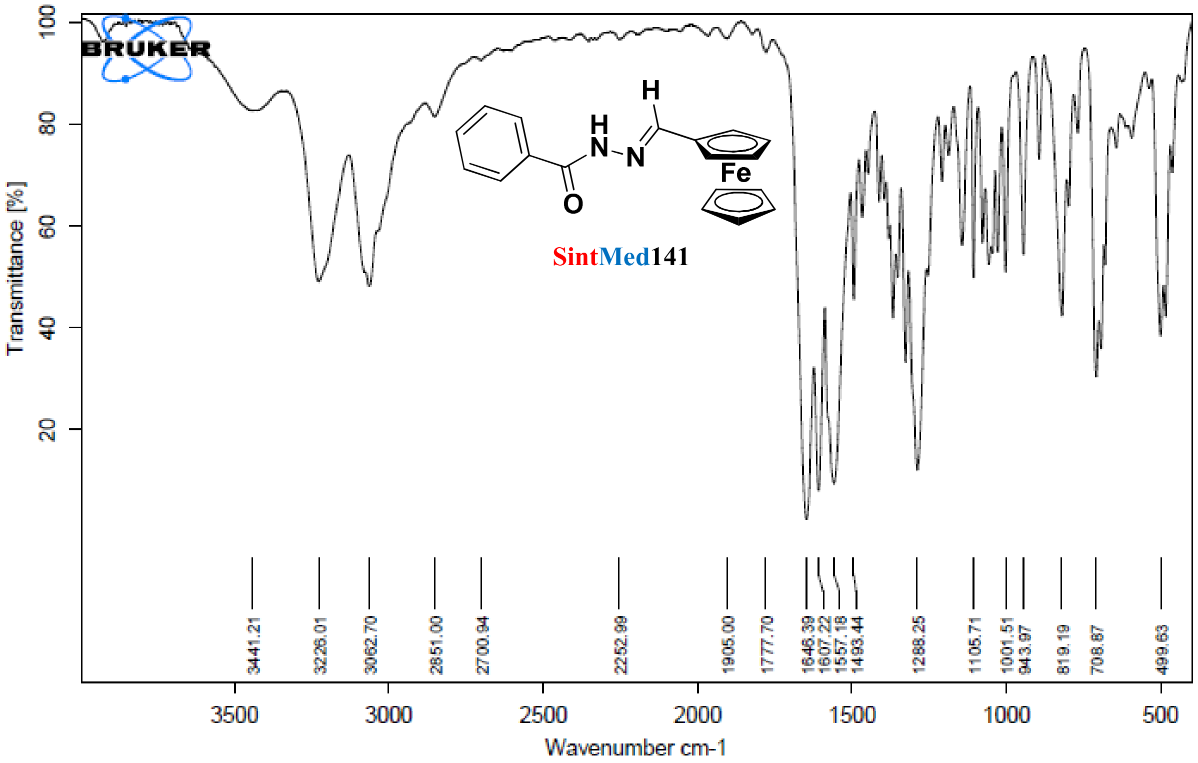

IR spectrum of SintMed141

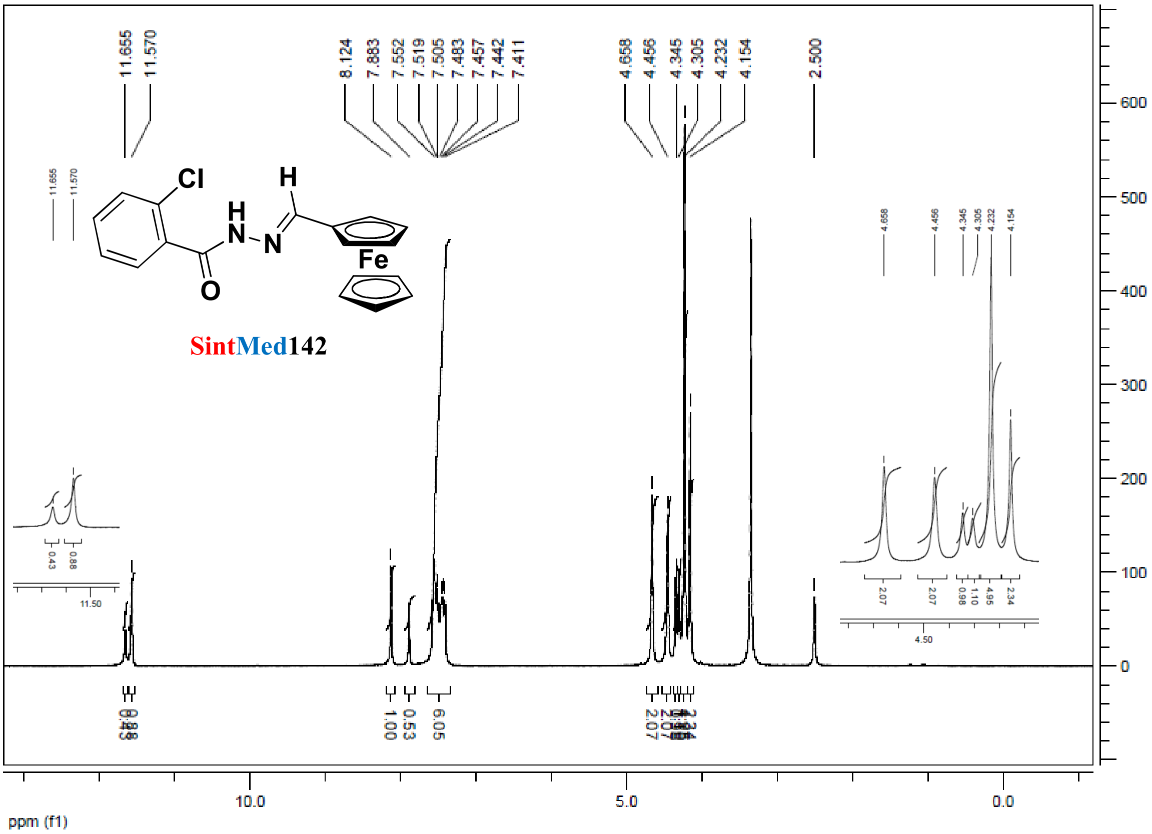

^{1}H NMR spectrum of SintMed142 (400 MHz, DMSO-d_6)

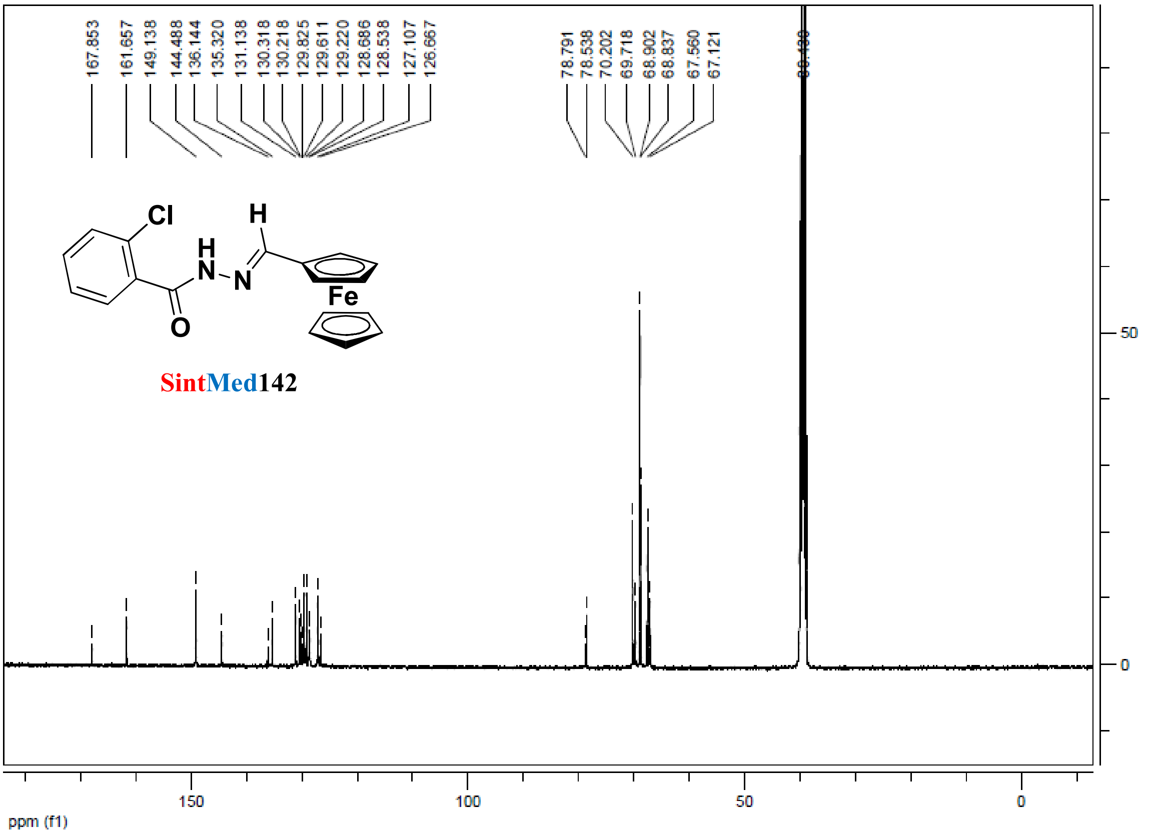

^{13}C NMR spectrum of SintMed142 (100 MHz, DMSO-d_6)

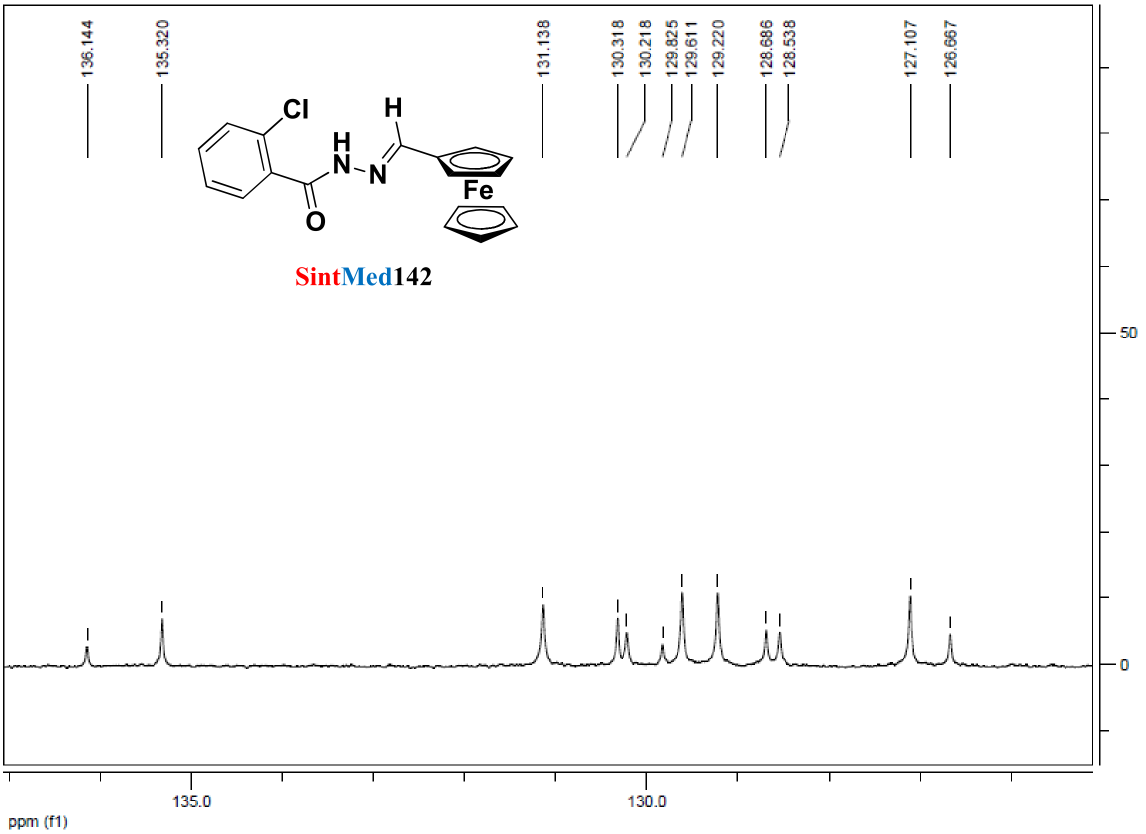

^{13}C NMR spectrum expansion of SintMed142 (100 MHz, DMSO-d_6)

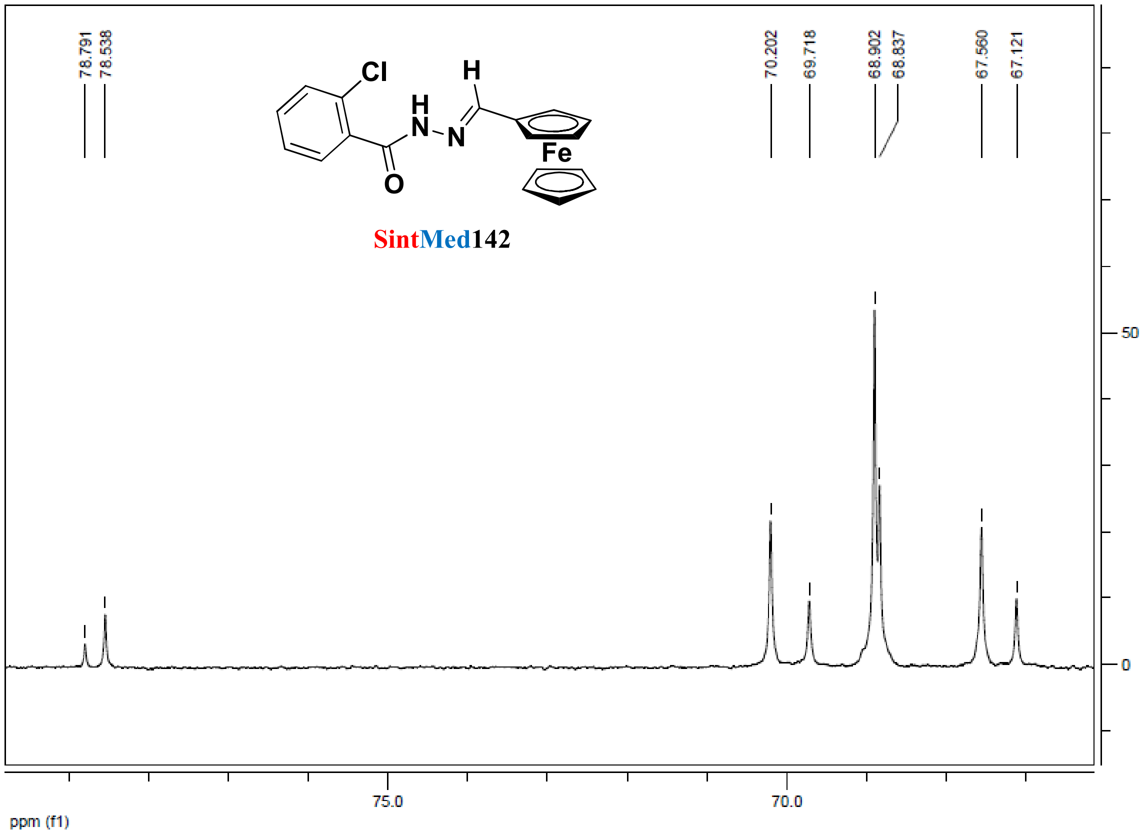

^{13}C NMR spectrum expansion of SintMed142 (100 MHz, DMSO-d_6)

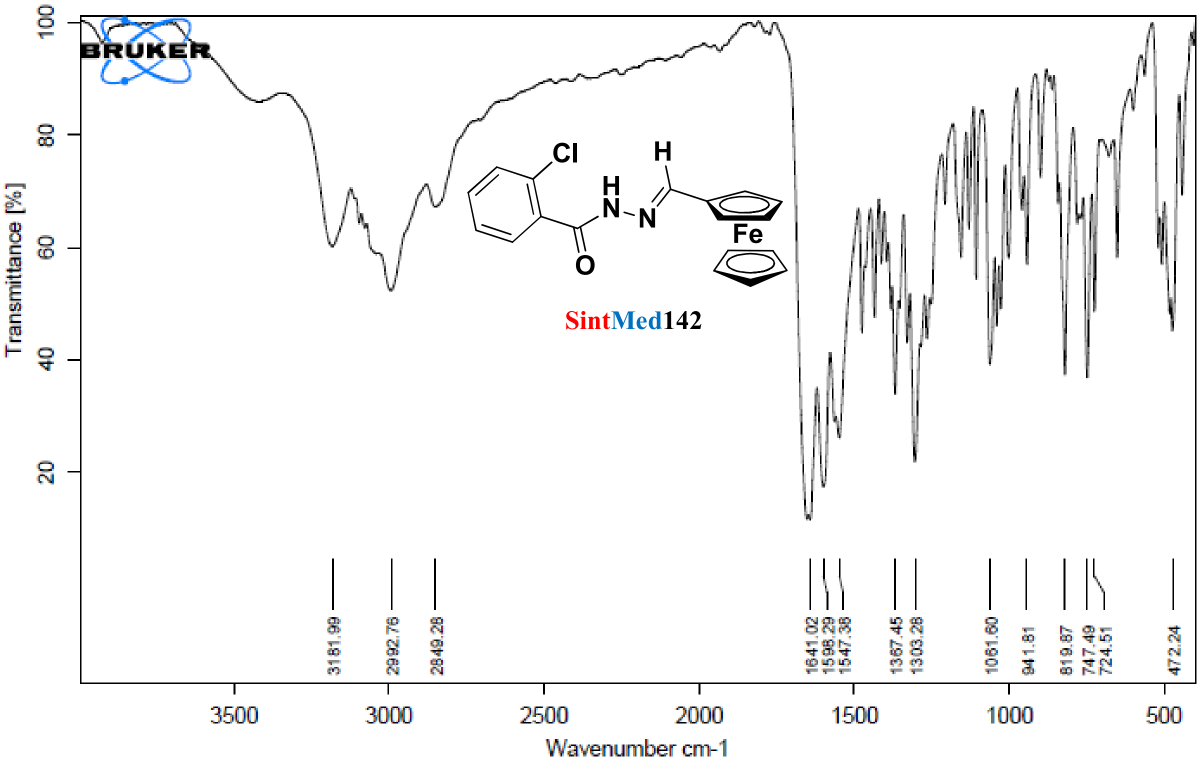

IR spectrum of SintMed142

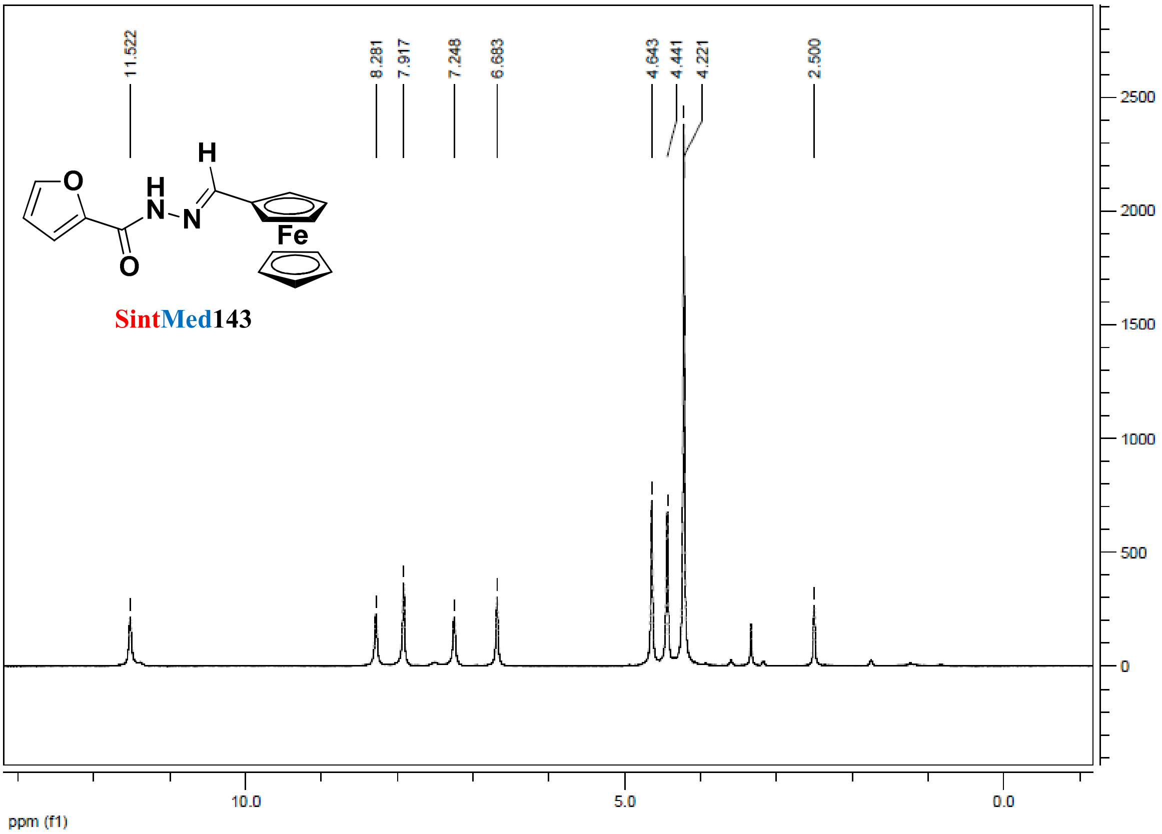

^{1}H NMR spectrum of SintMed143 (300 MHz, DMSO-d_6)

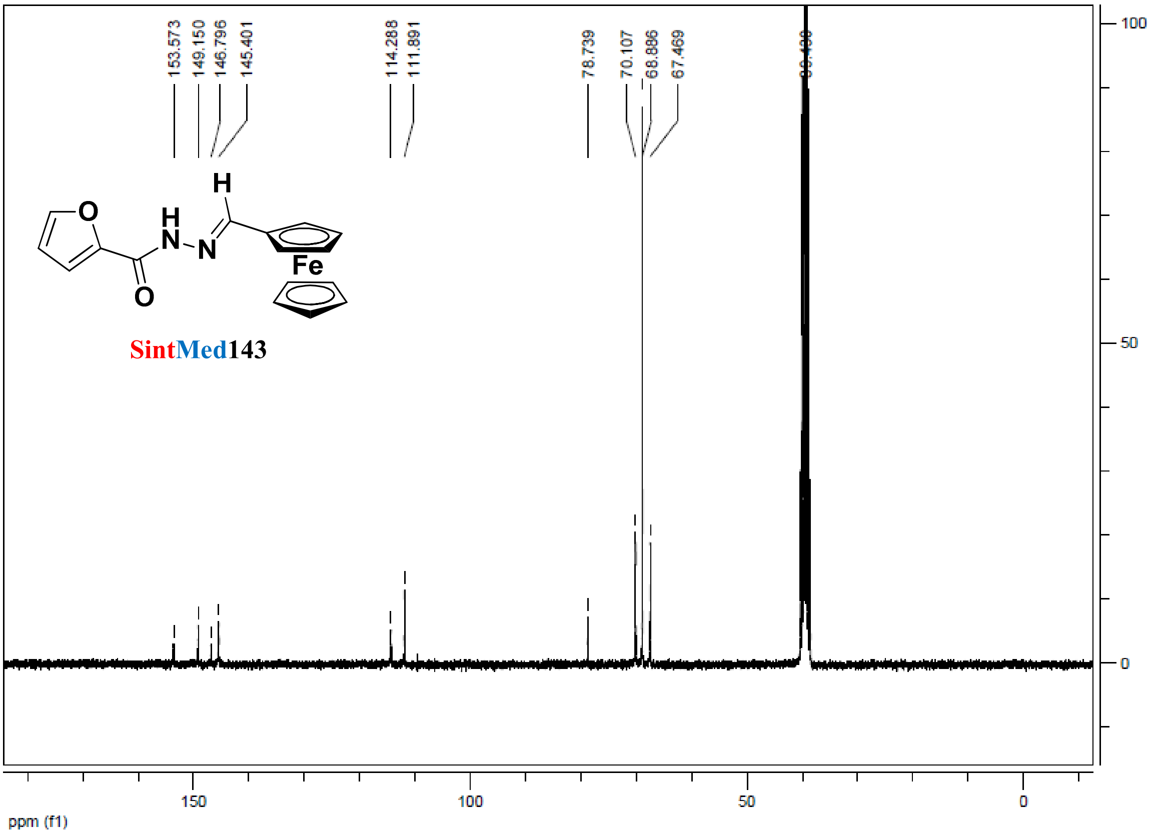

^{13}C NMR spectrum of SintMed143 (75 MHz, DMSO-d_6)

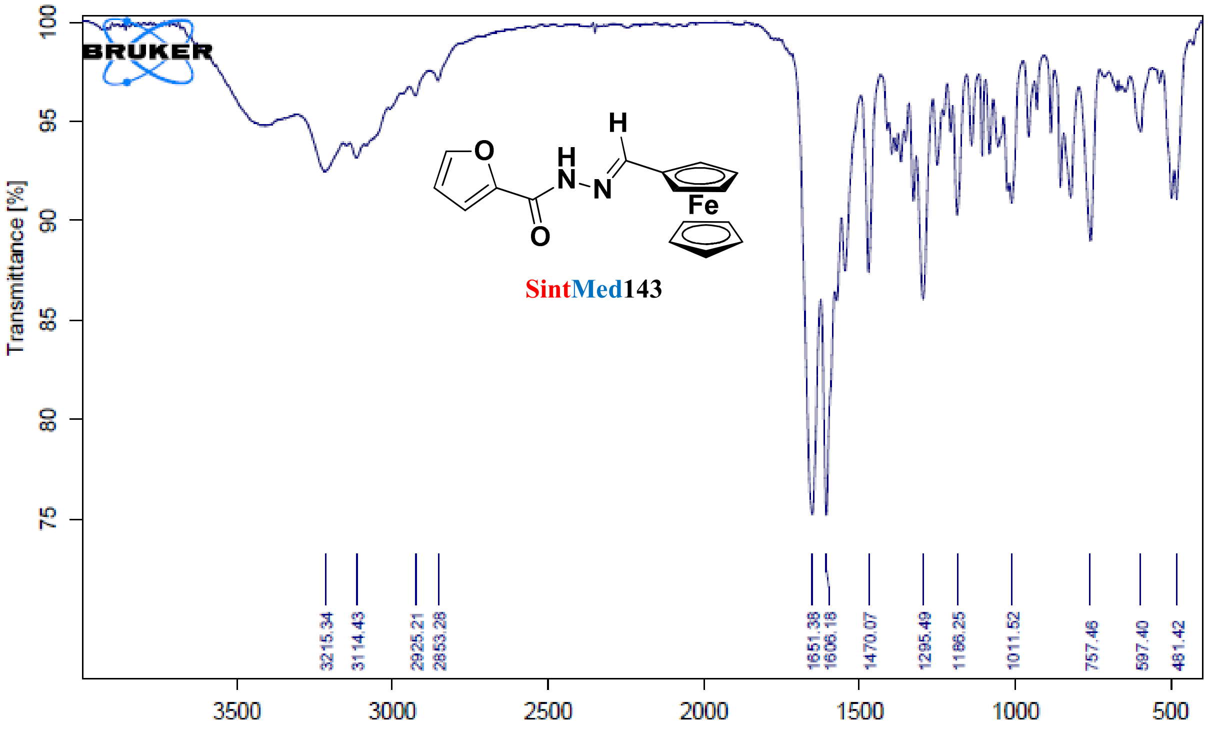

IR spectrum of SintMed143

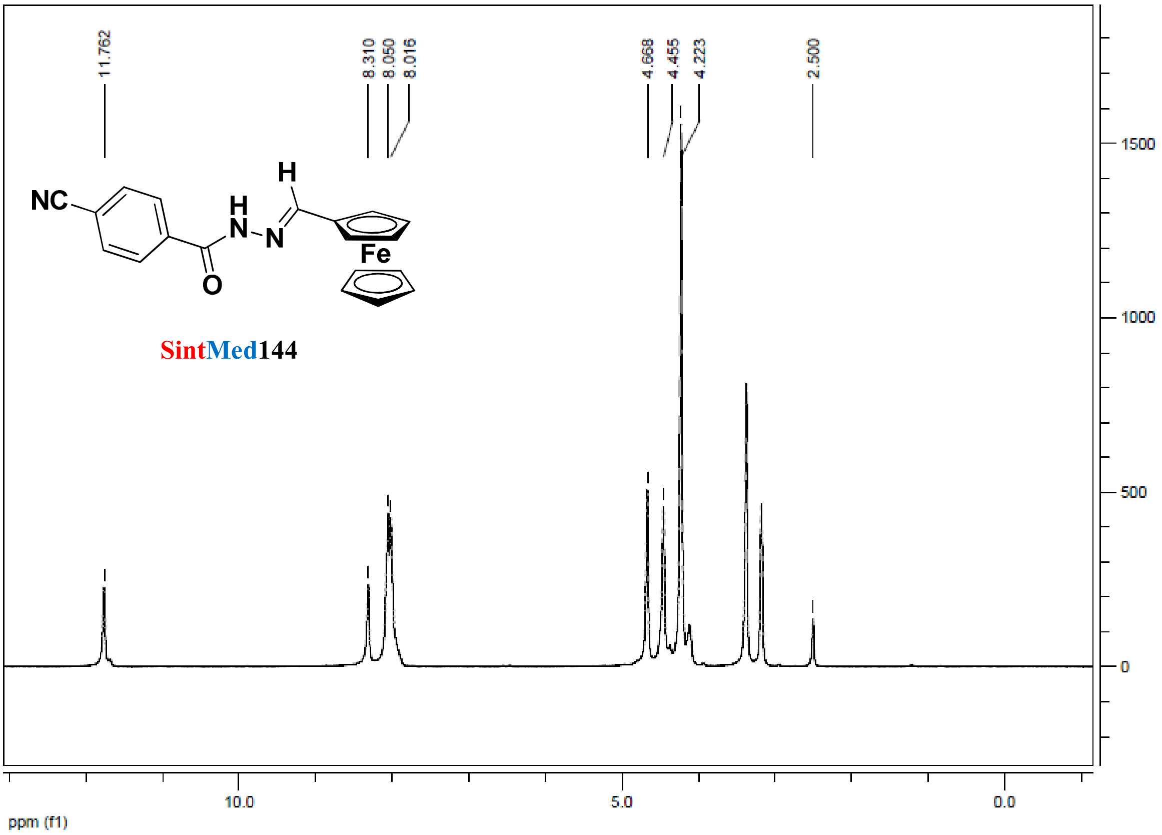

^{1}H NMR spectrum of SintMed144 (300 MHz, DMSO-d_6)

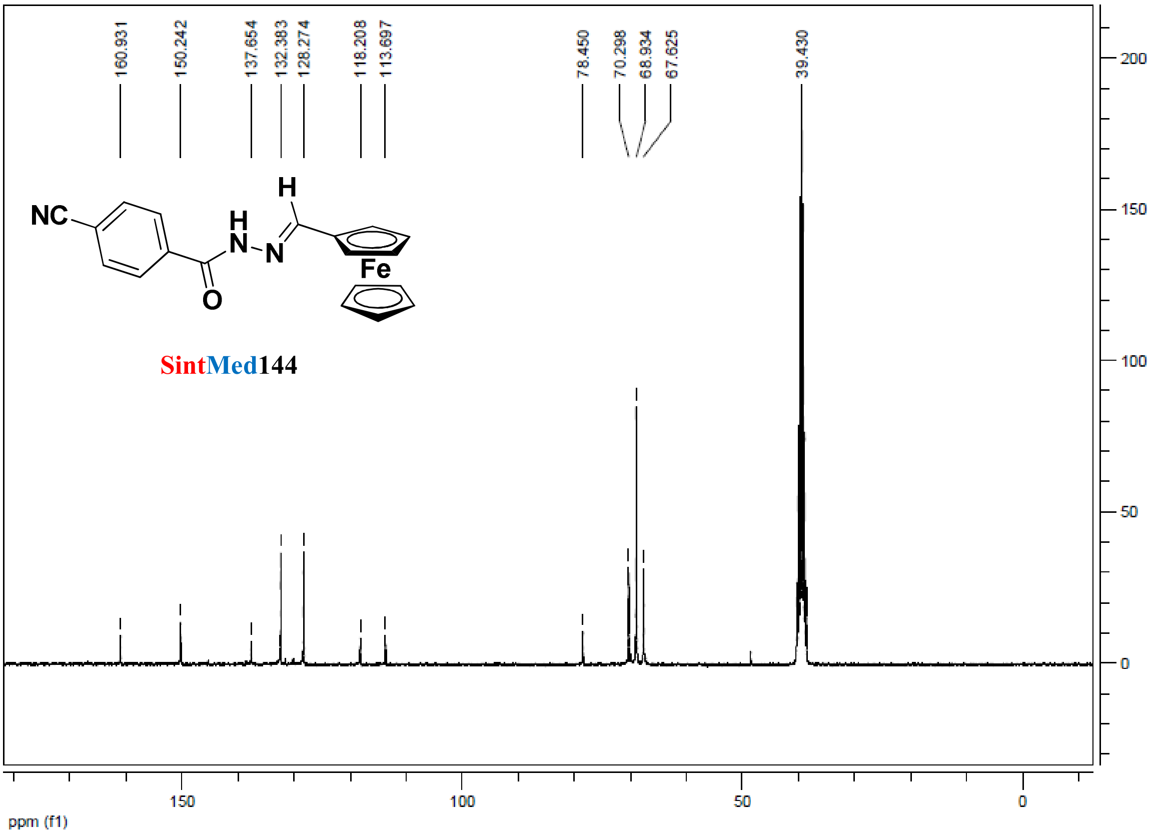

^{13}C NMR spectrum of SintMed144 (75 MHz, DMSO-d_6)

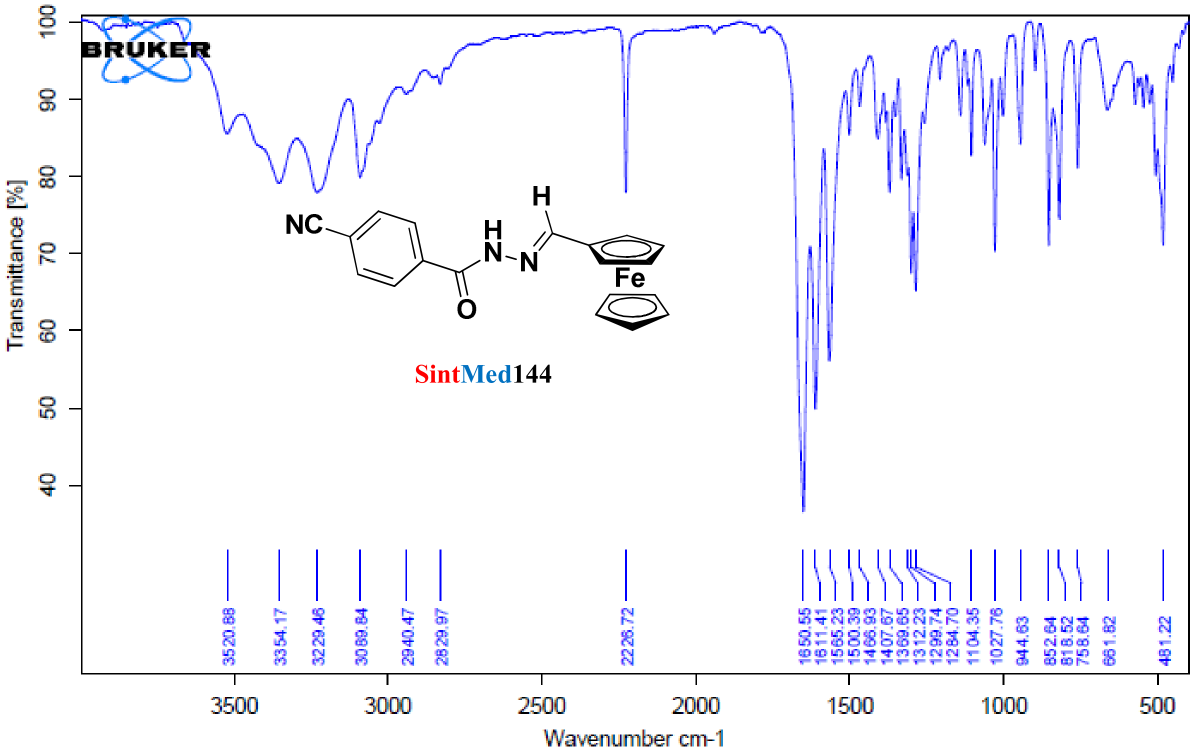

IR spectrum of SintMed144

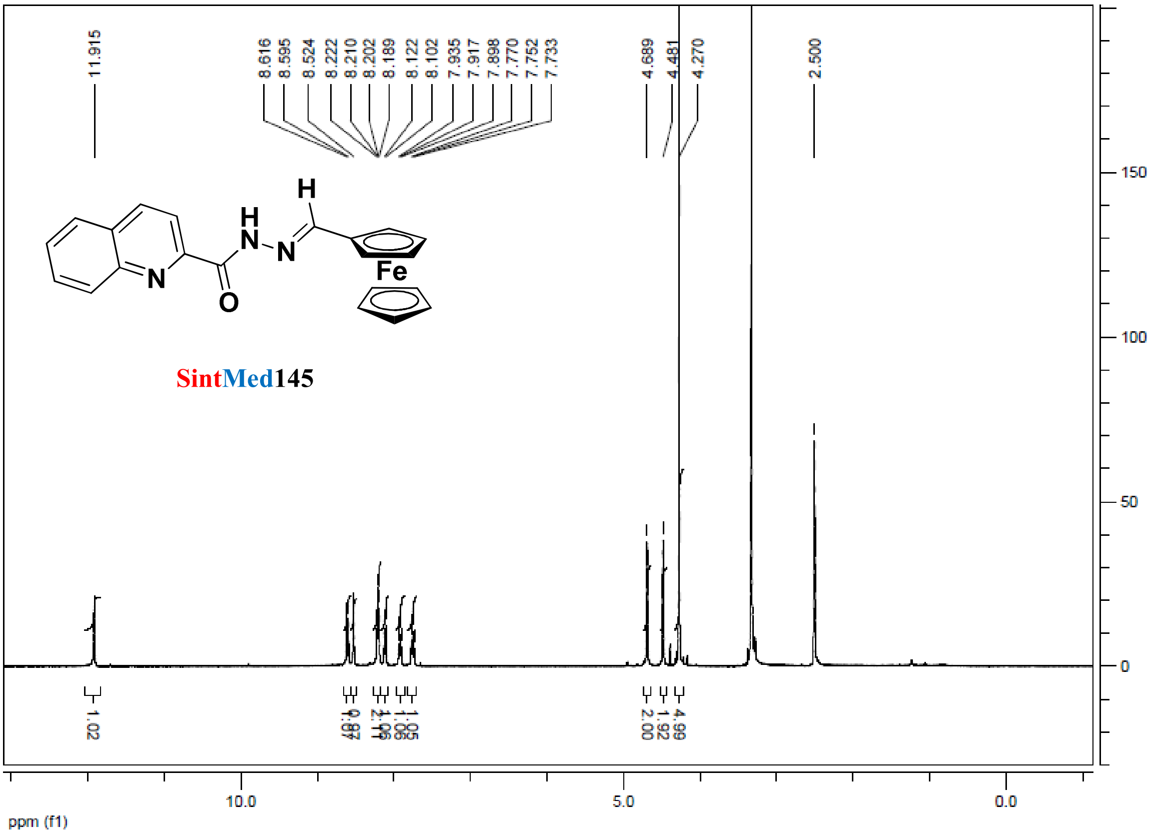

^{1}H NMR spectrum of SintMed145 (400 MHz, DMSO-d_6)

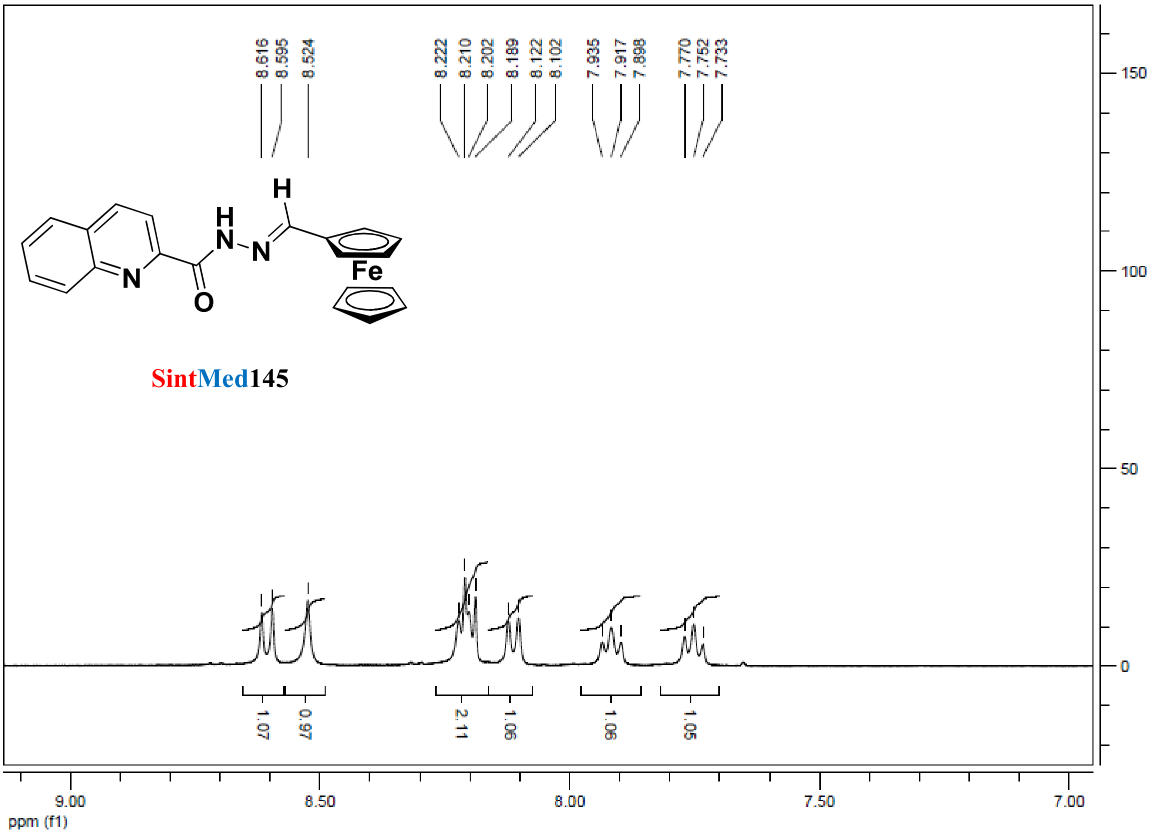

^{1}H NMR spectrum expansion of SintMed145 (400 MHz, DMSO-d_6)

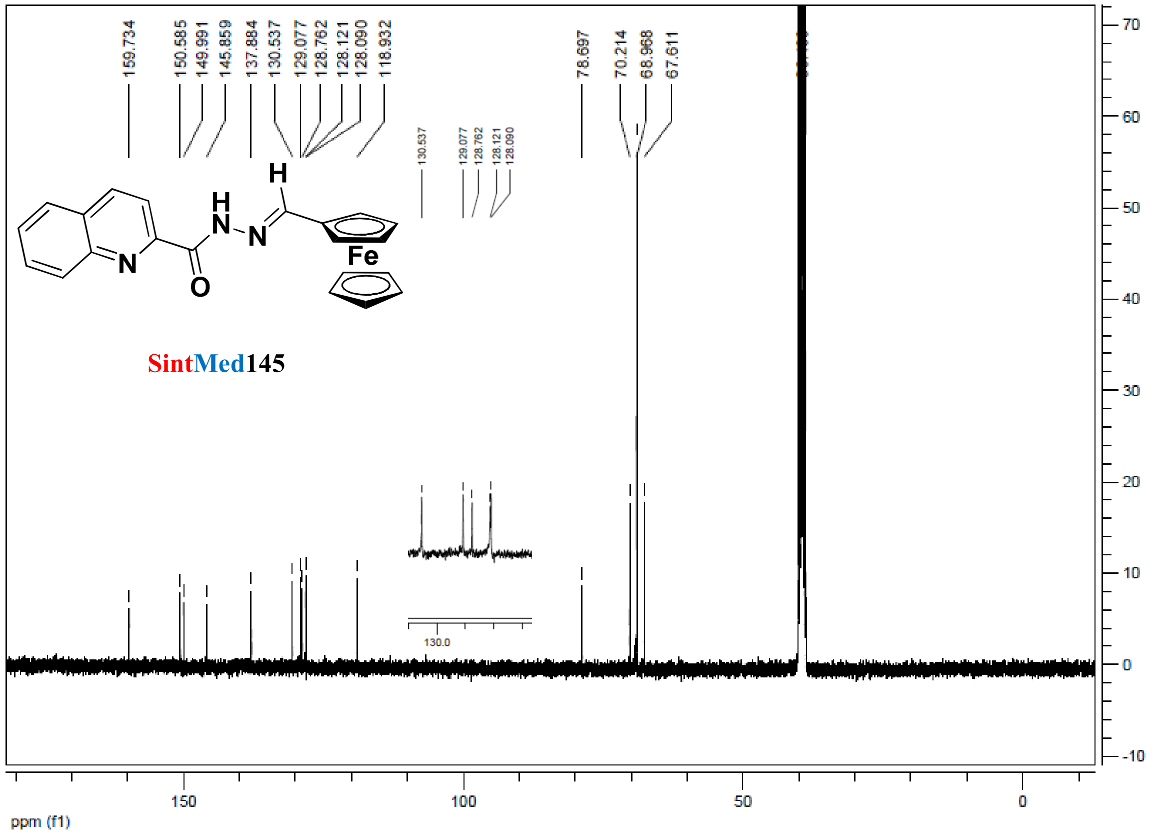

^{13}C NMR spectrum of SintMed145 (100 MHz, DMSO-d_6)

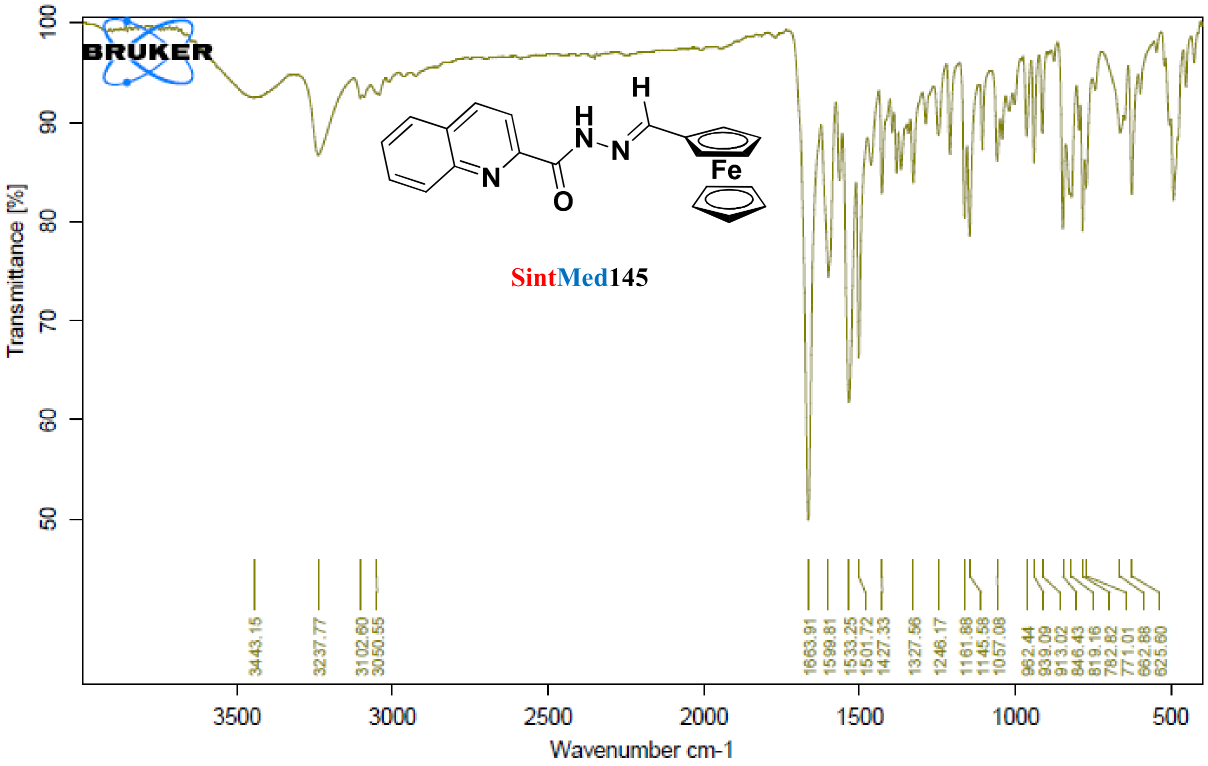

IR spectrum of SintMed145

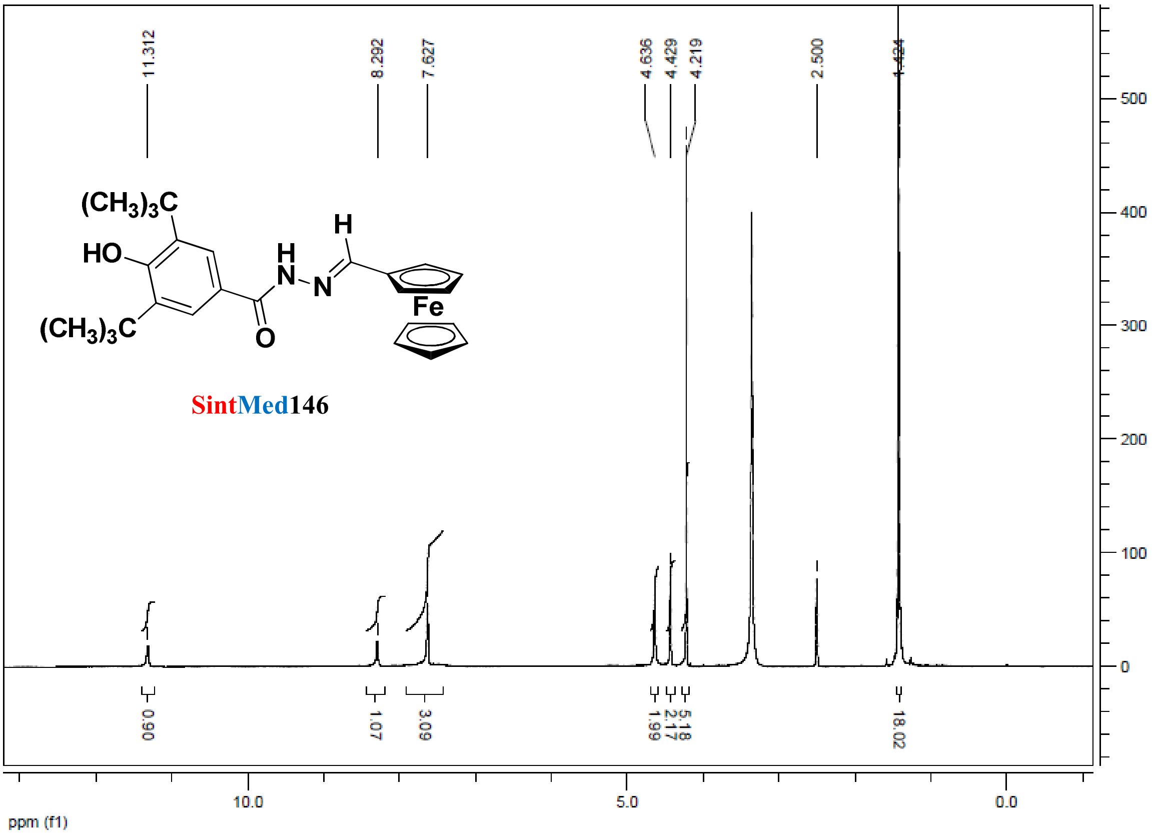

^{1}H NMR spectrum of SintMed146 (400 MHz, DMSO-d_6)

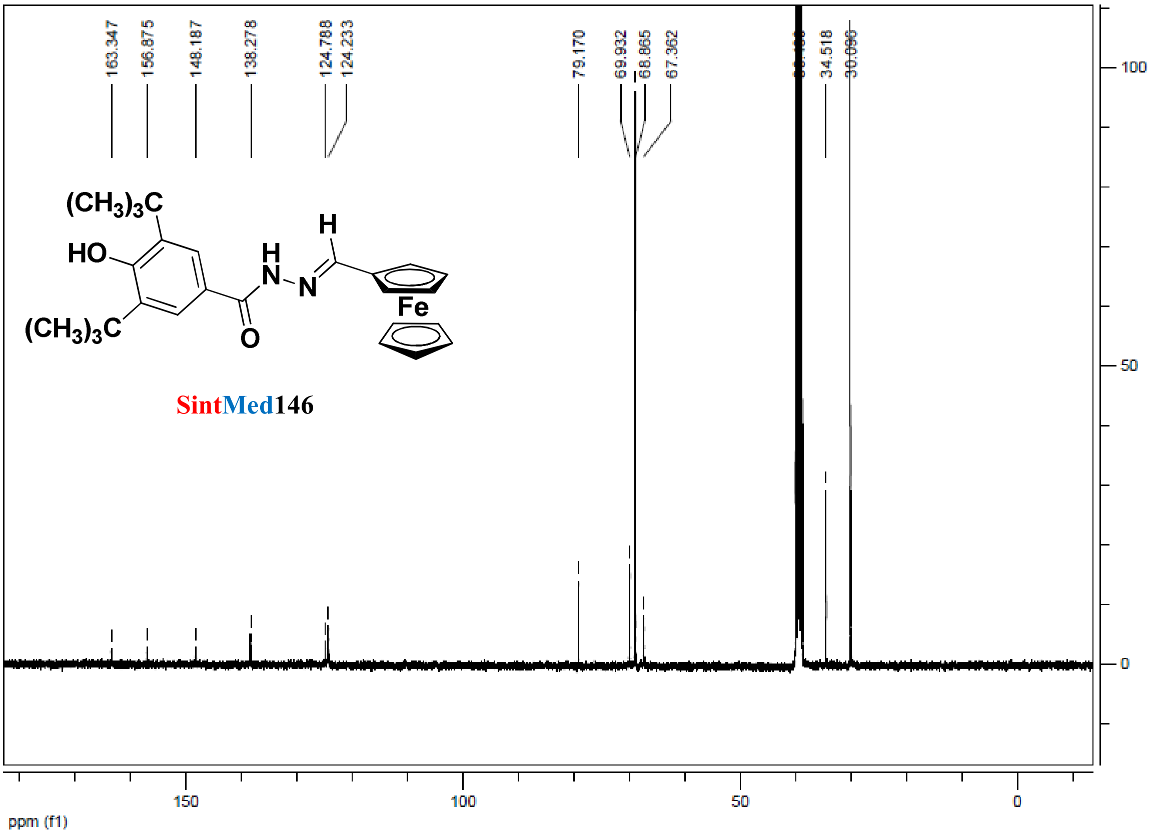

^{13}C NMR spectrum of SintMed146 (100 MHz, DMSO-d_6)

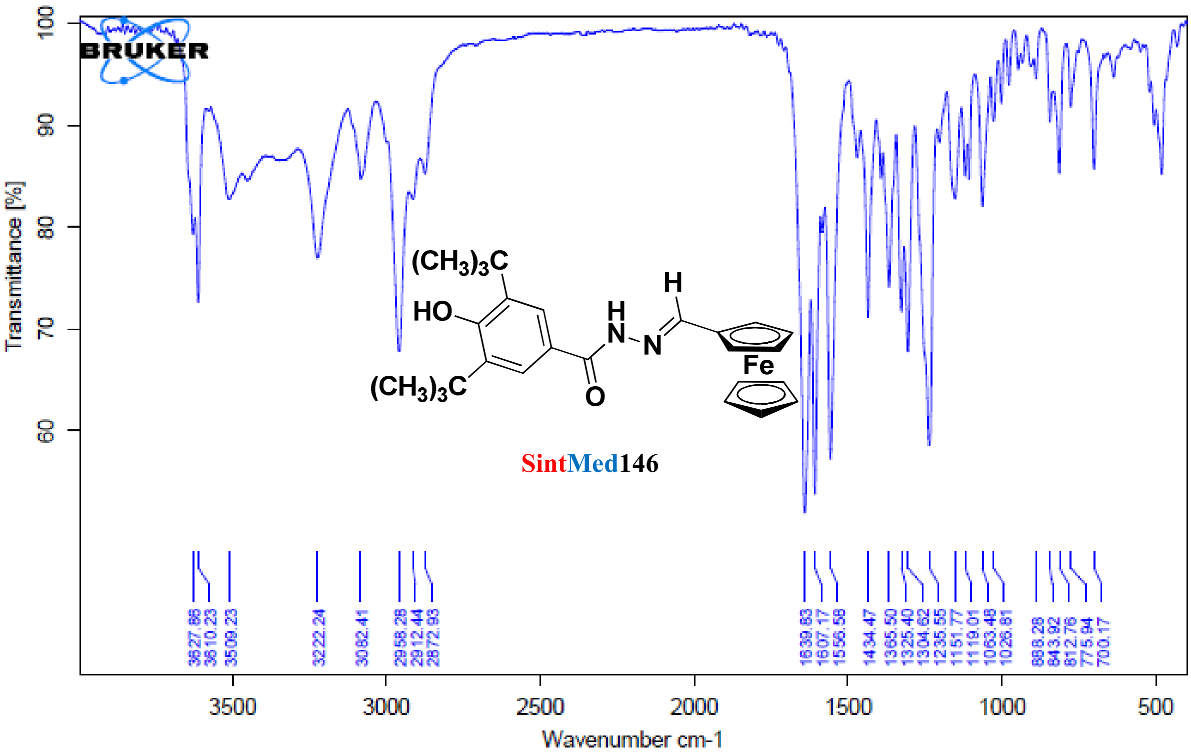

IR spectrum of SintMed146

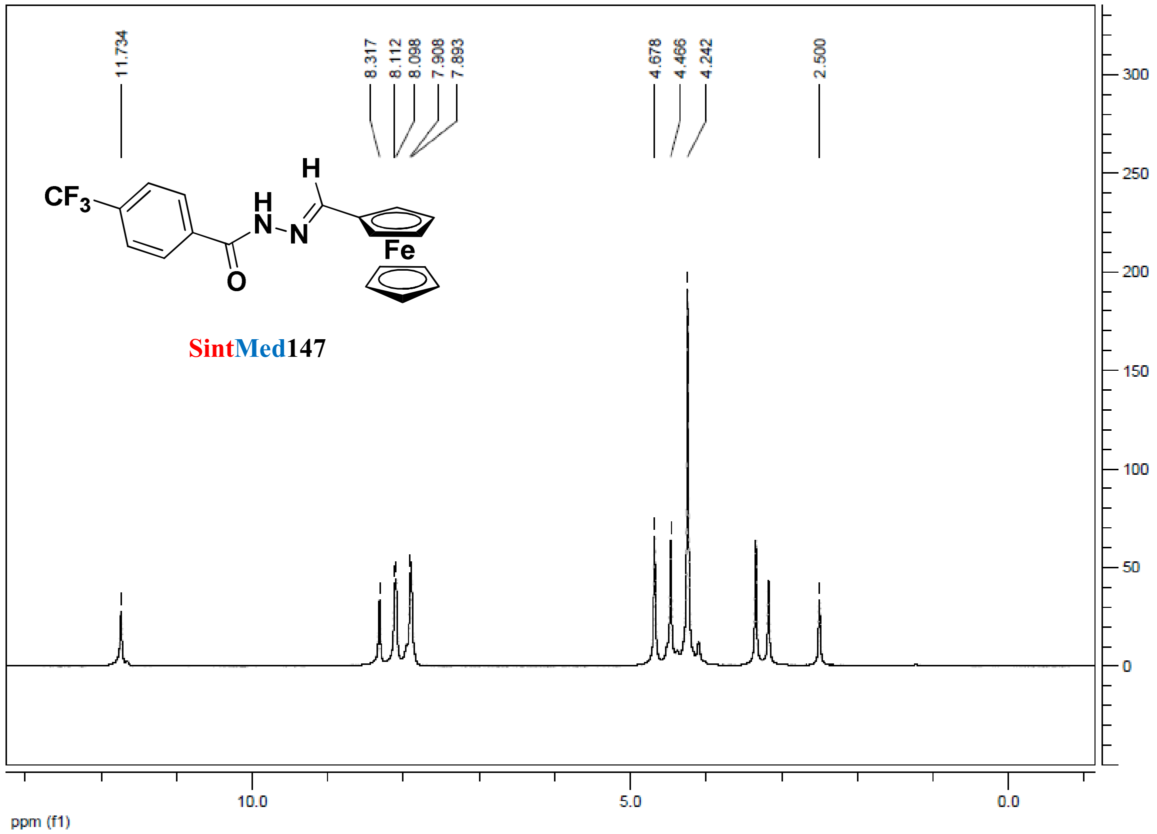

^{1}H NMR spectrum of SintMed147 (400 MHz, DMSO-d_6)

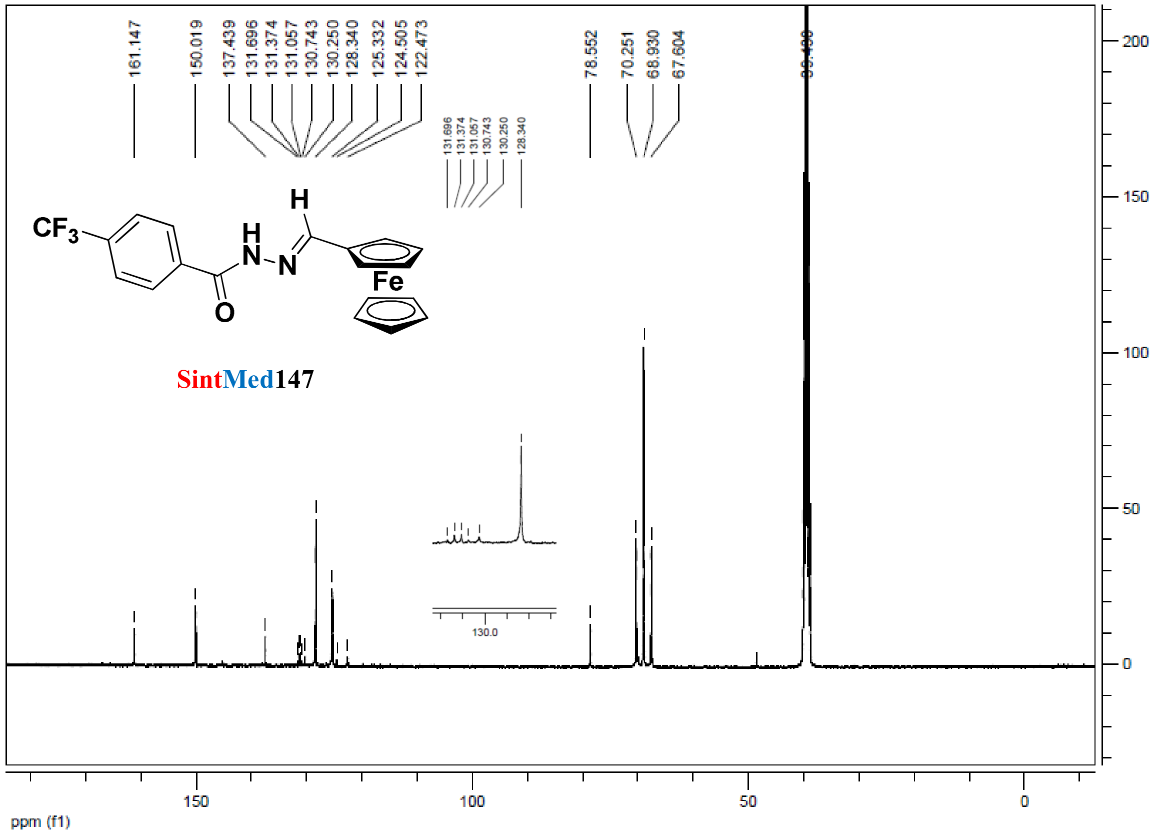

^{13}C NMR spectrum of SintMed147 (100 MHz, DMSO-d_6)

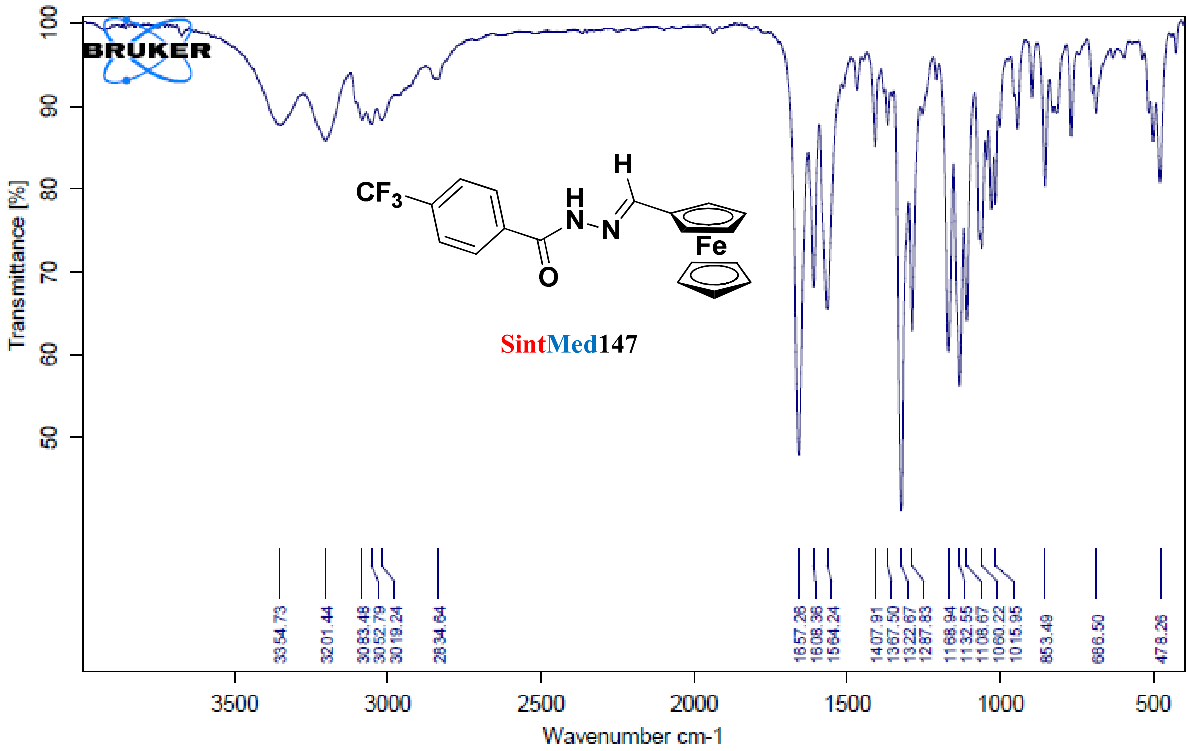

IR spectrum of SintMed147

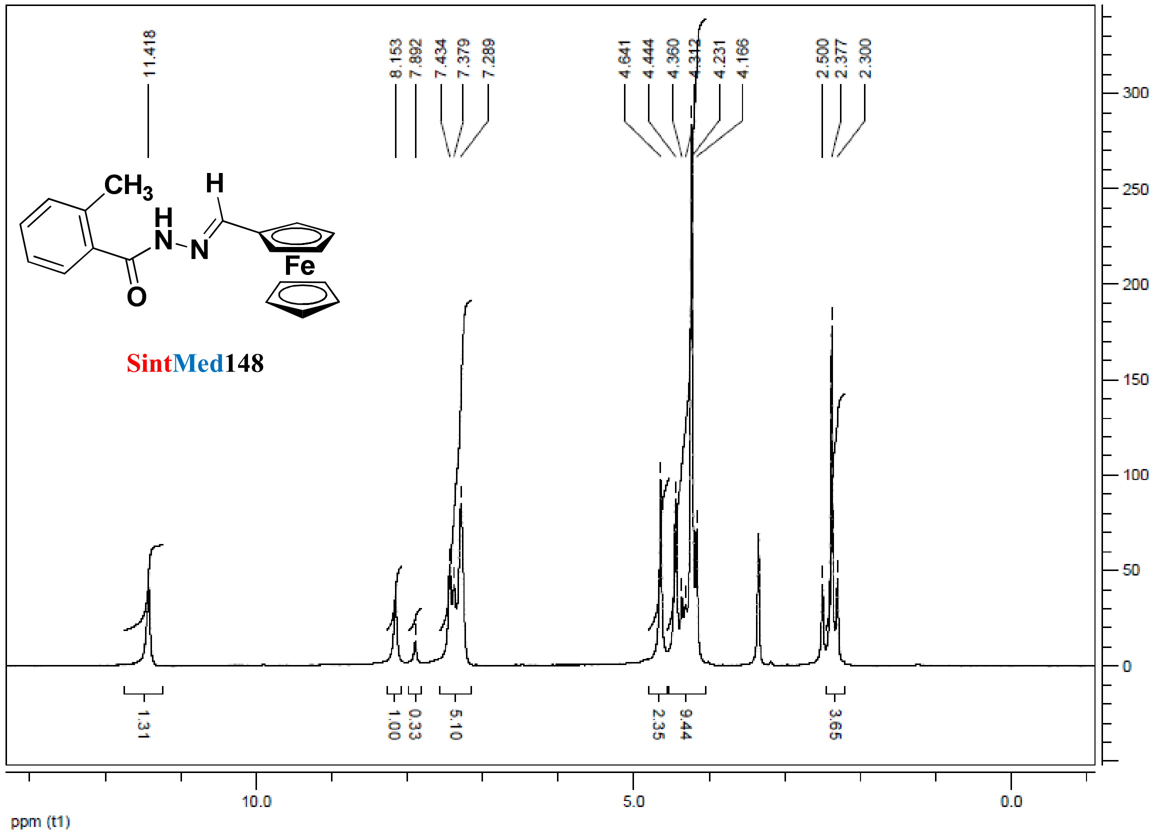

^{1}H NMR spectrum of SintMed148 (400 MHz, DMSO-d_6)

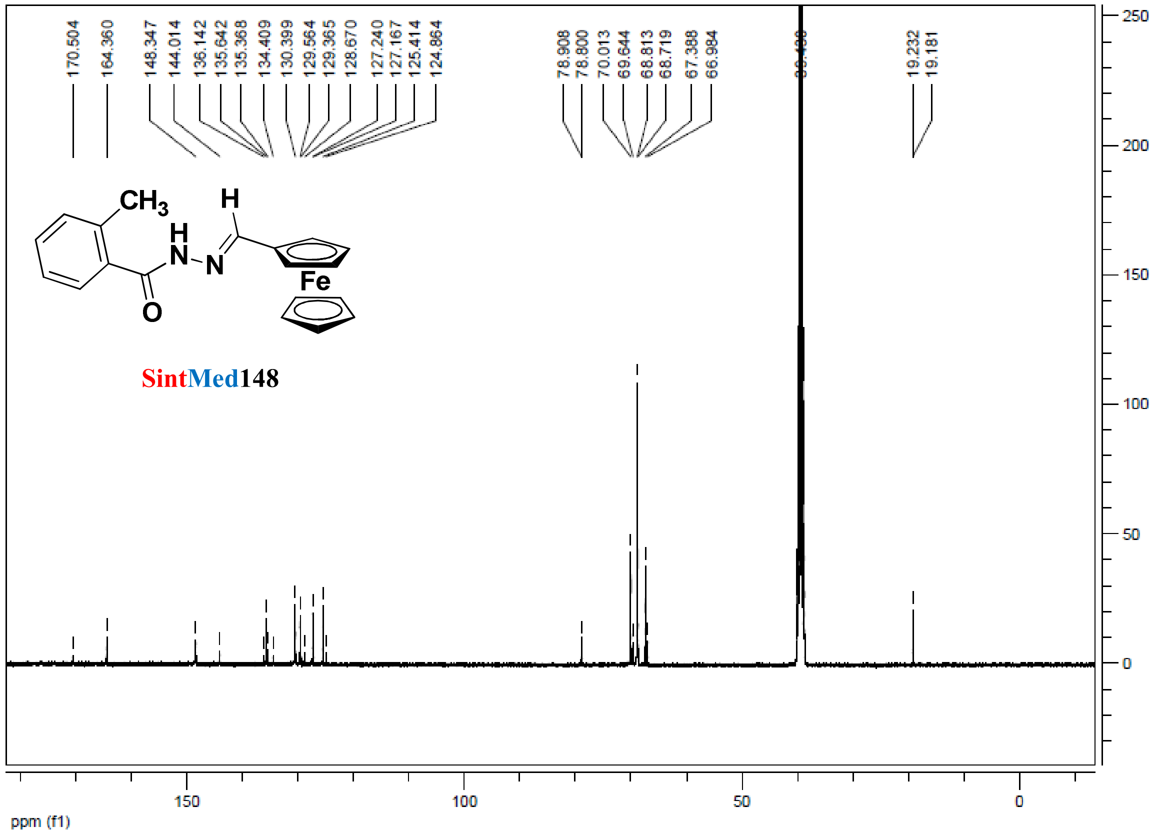

^{13}C NMR spectrum of SintMed148 (100 MHz, DMSO-d_6)

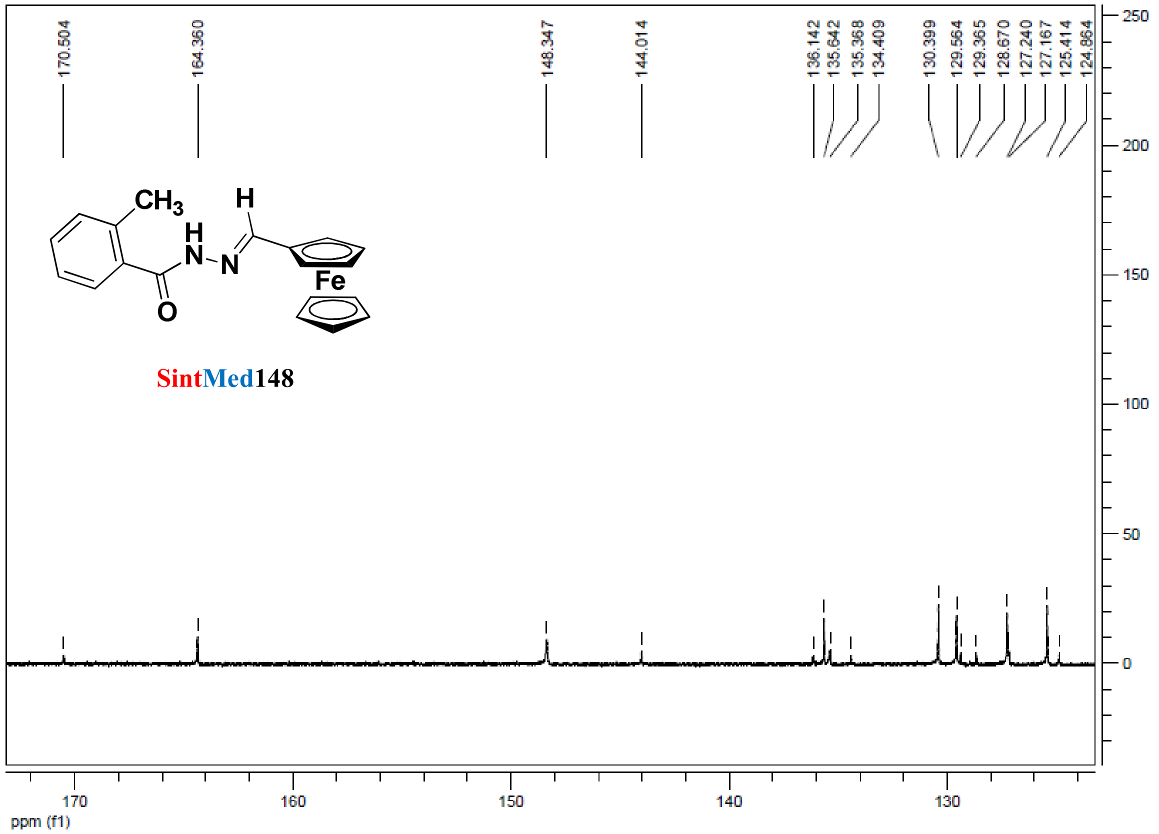

^{13}C NMR spectrum expansion of SintMed148 (100 MHz, DMSO-d_6)

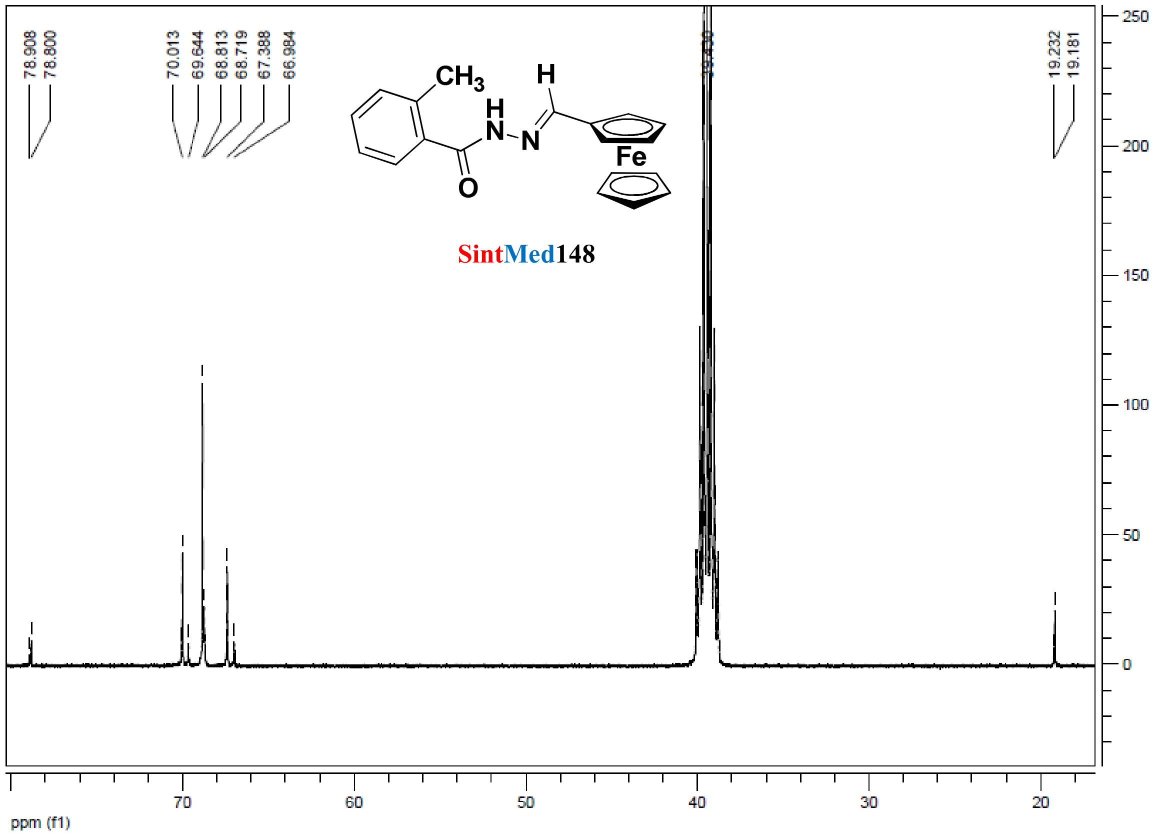

^{13}C NMR spectrum expansion of SintMed148 (100 MHz, DMSO-d_6)

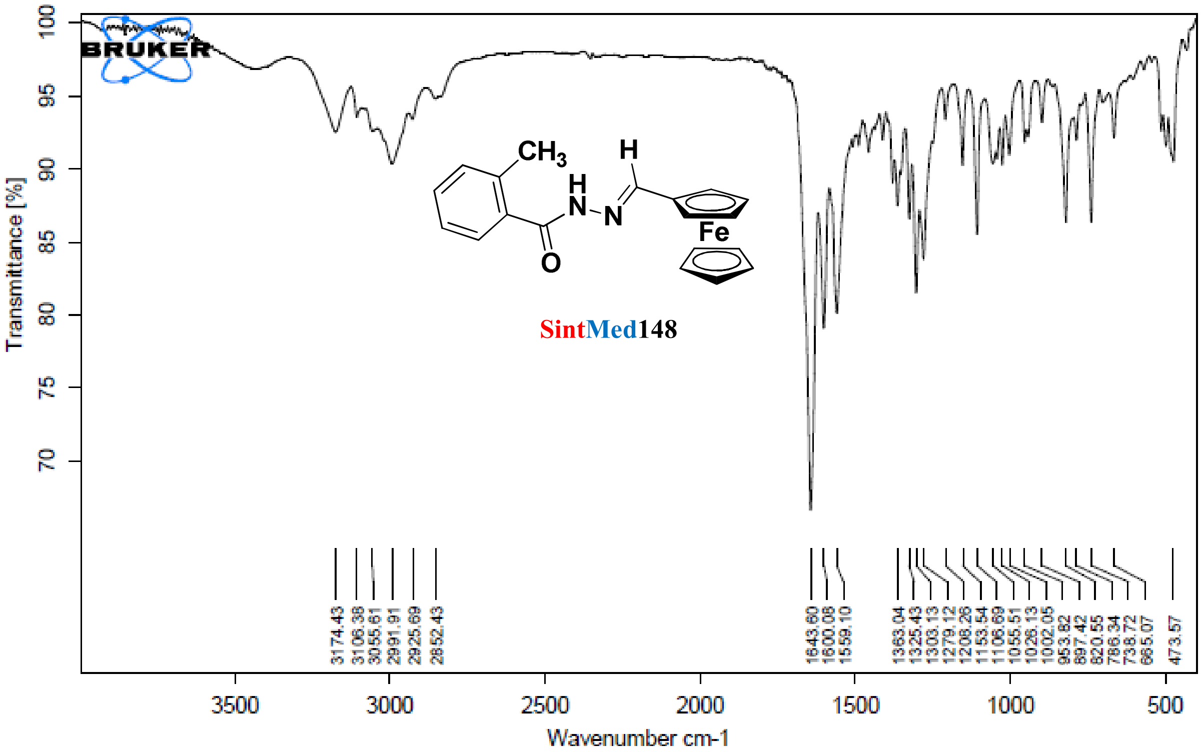

IR spectrum of SintMed148

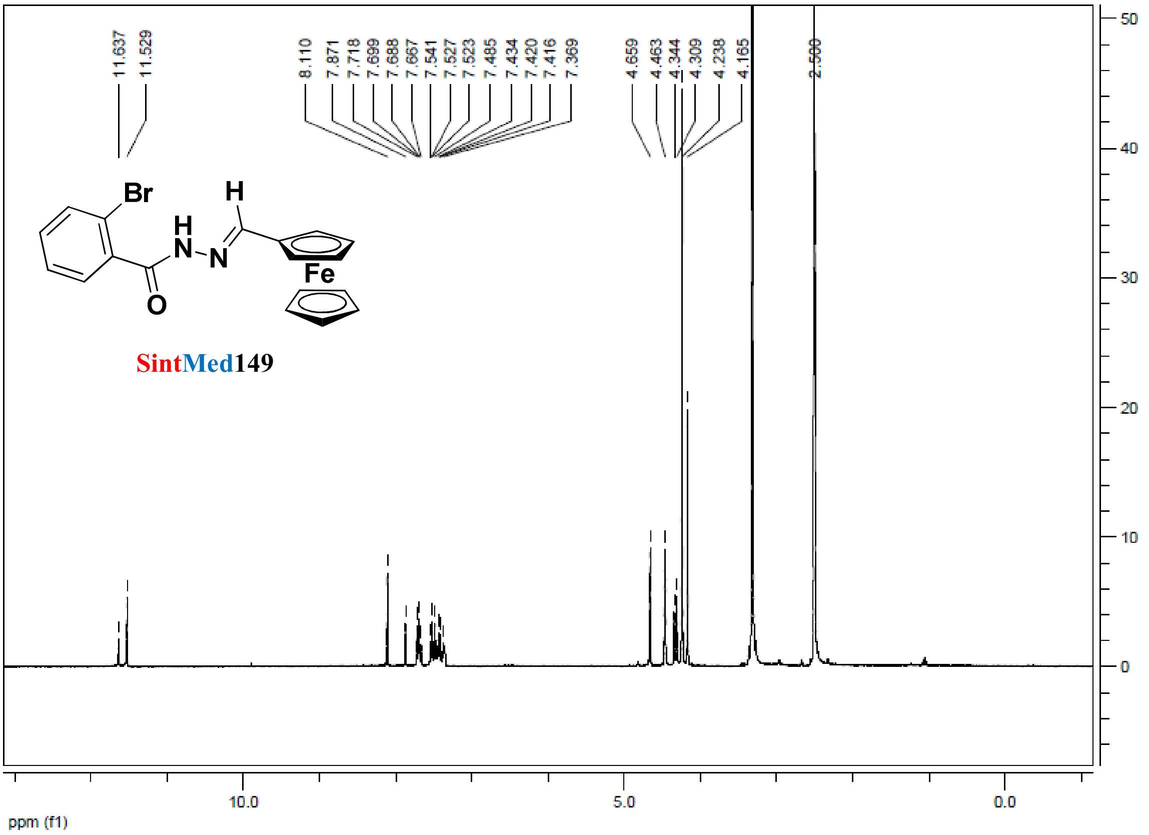

^{1}H NMR spectrum of SintMed149 (400 MHz, DMSO-d_6)

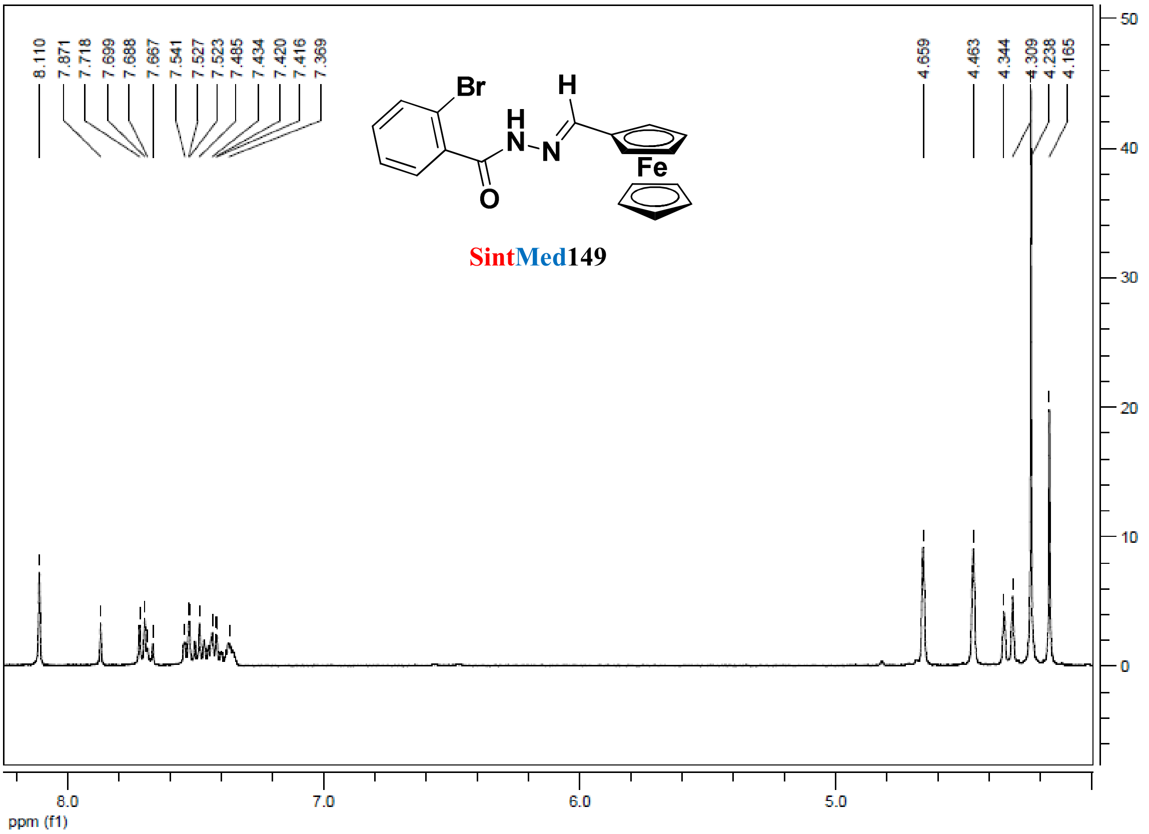

^{1}H NMR spectrum expansion of SintMed149 (400 MHz, DMSO-d_6)

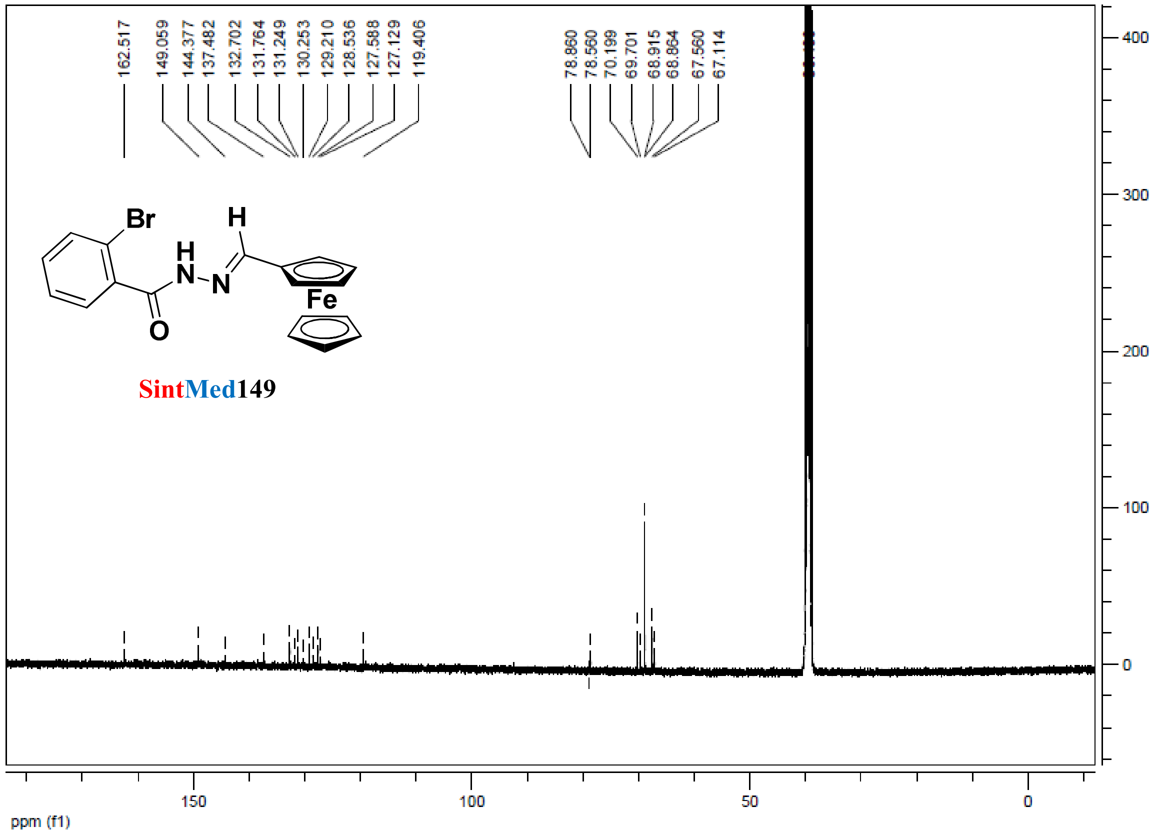

^{13}C NMR spectrum of SintMed149 (100 MHz, DMSO-d_6)

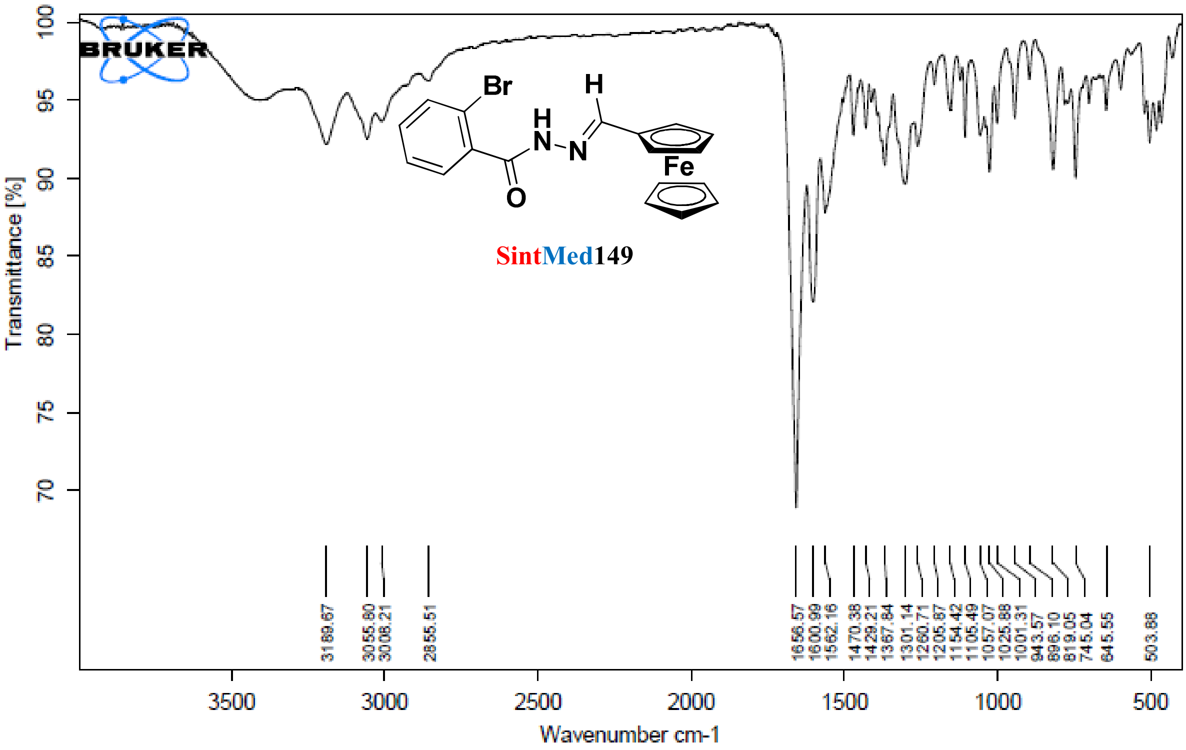

IR spectrum of SintMed149

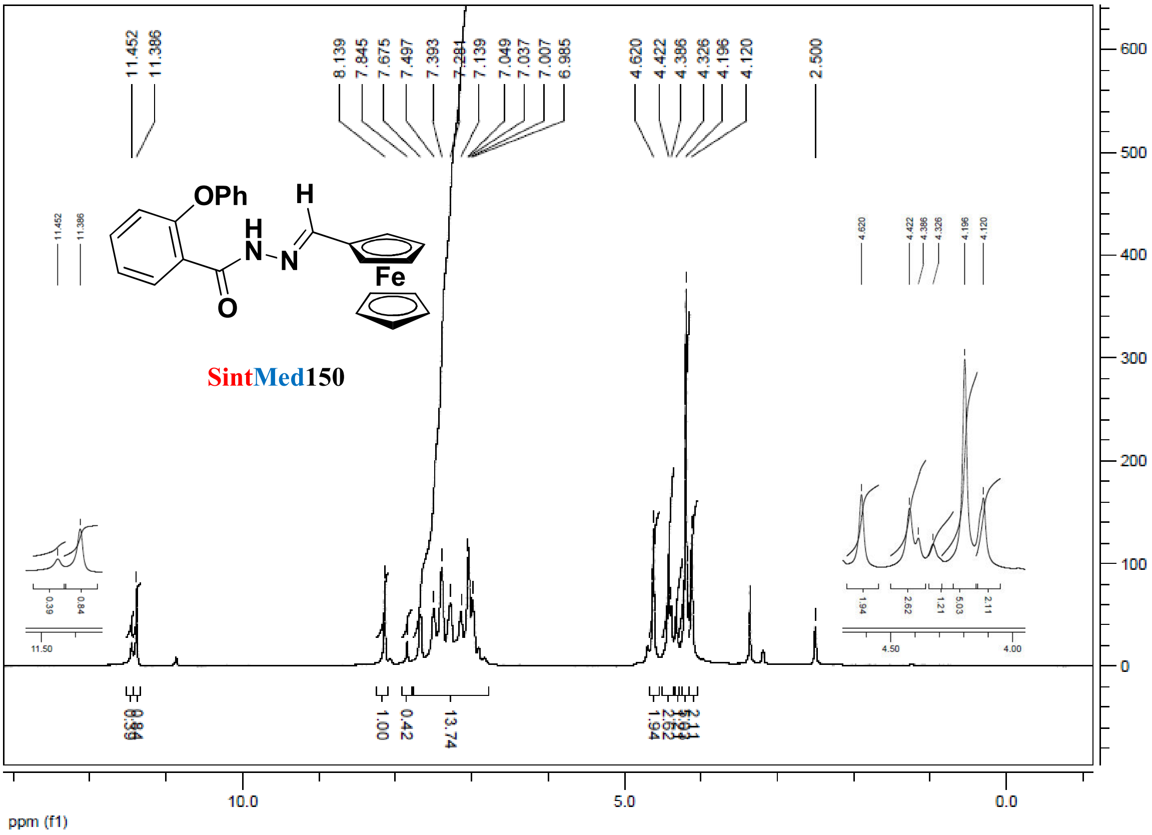

^{1}H NMR spectrum of SintMed150 (400 MHz, DMSO-d_6)

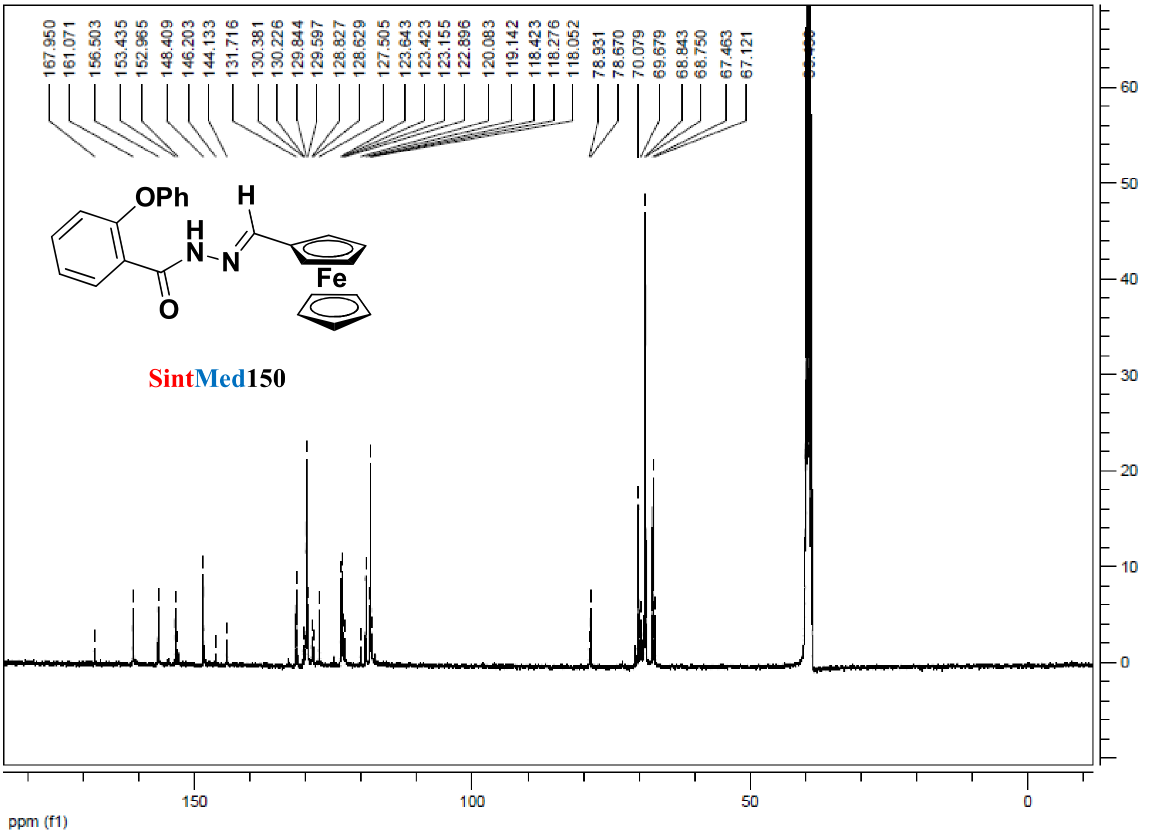

^{13}C NMR spectrum of SintMed150 (100 MHz, DMSO-d_6)

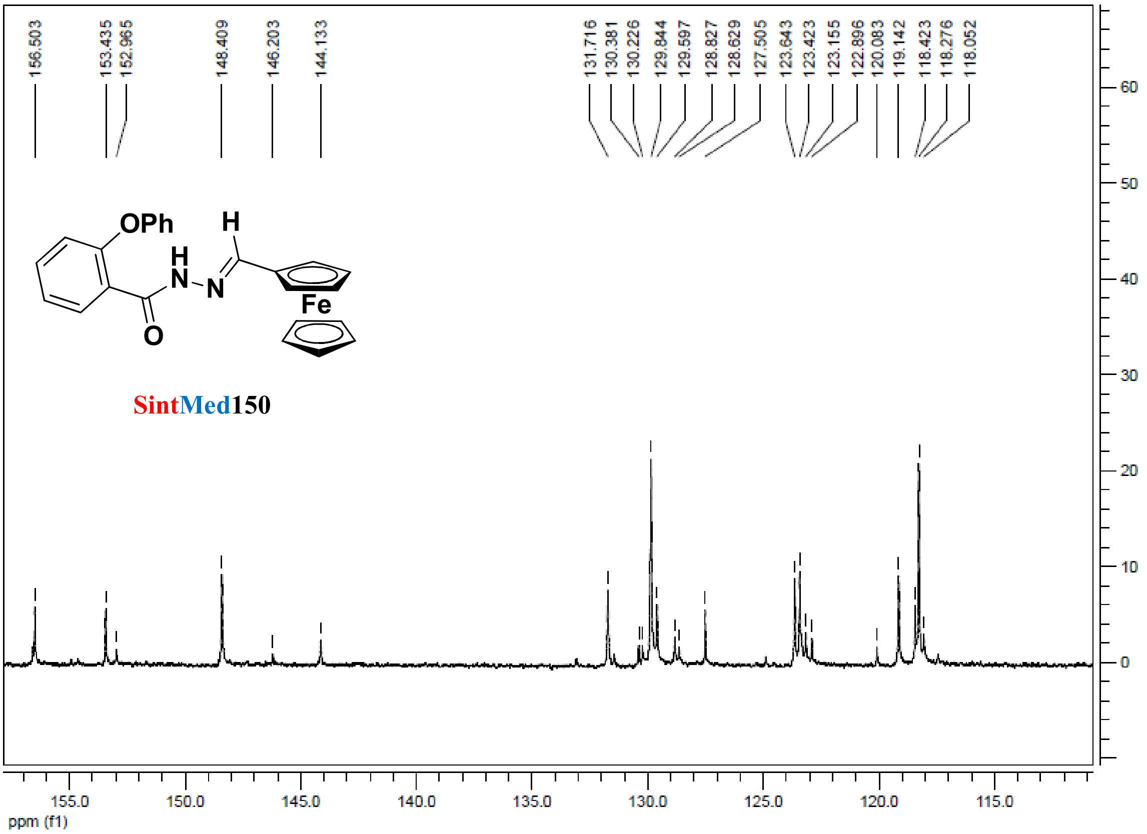

^{13}C NMR spectrum expansion of SintMed150 (100 MHz, DMSO-d_6)

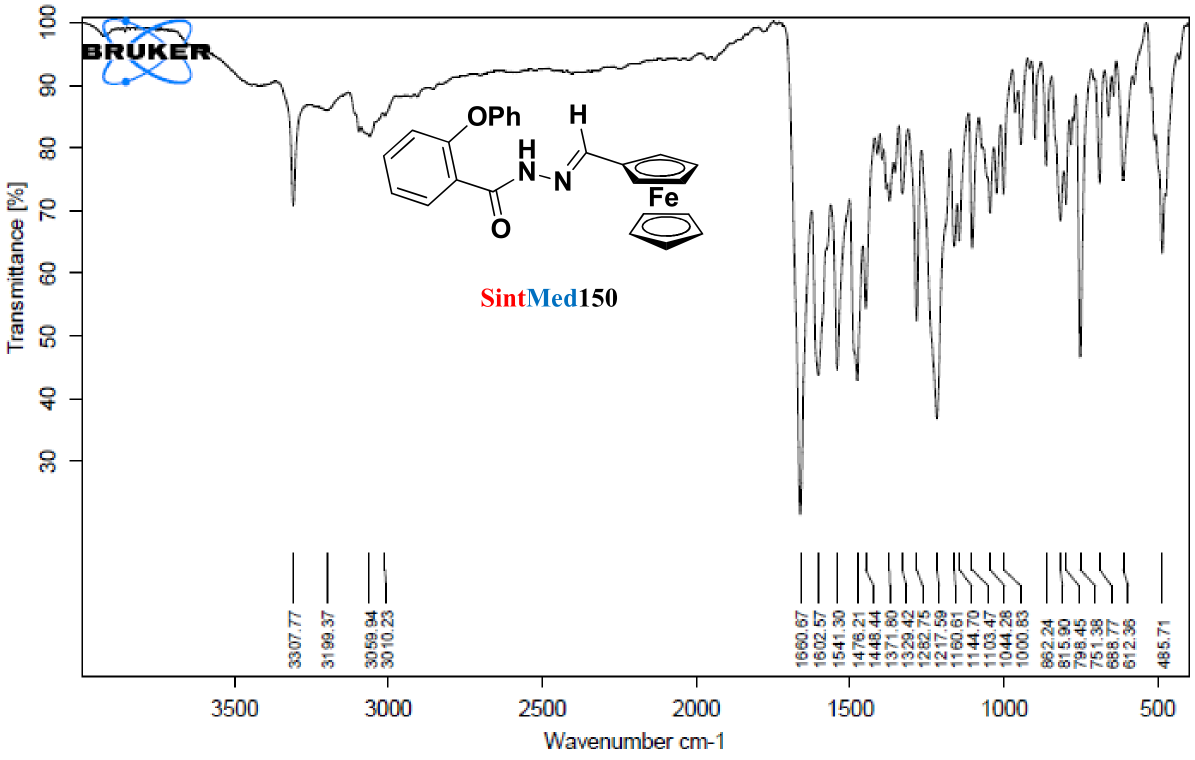

IR spectrum of SintMed150

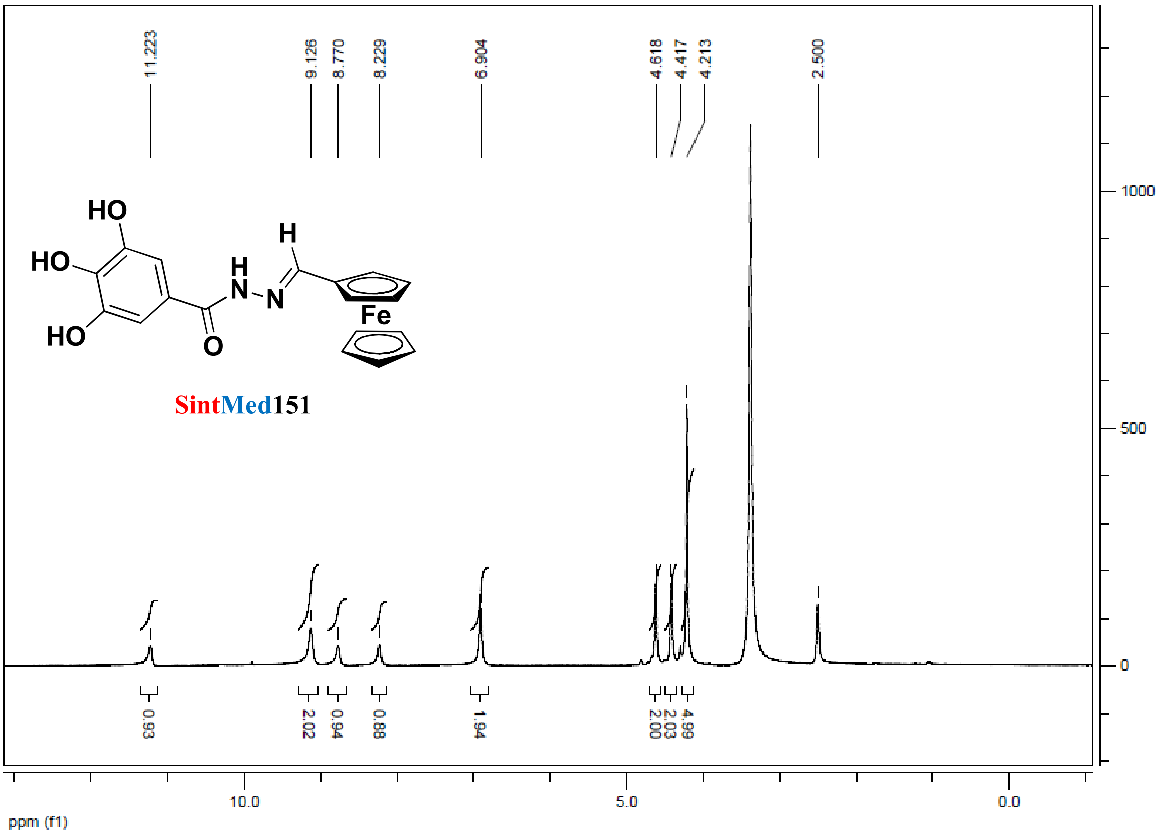

^{1}H NMR spectrum of SintMed151 (300 MHz, DMSO-d_6)

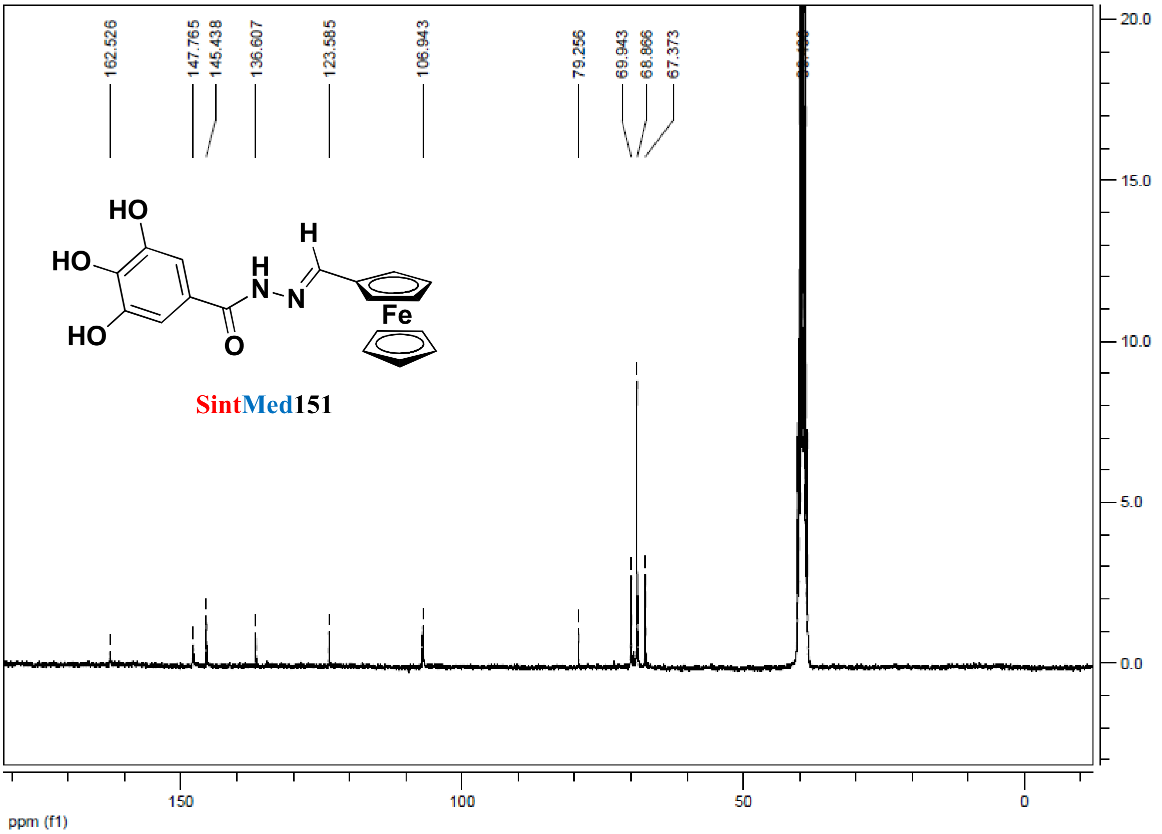

^{13}C NMR spectrum of SintMed151 (75 MHz, DMSO-d_6)

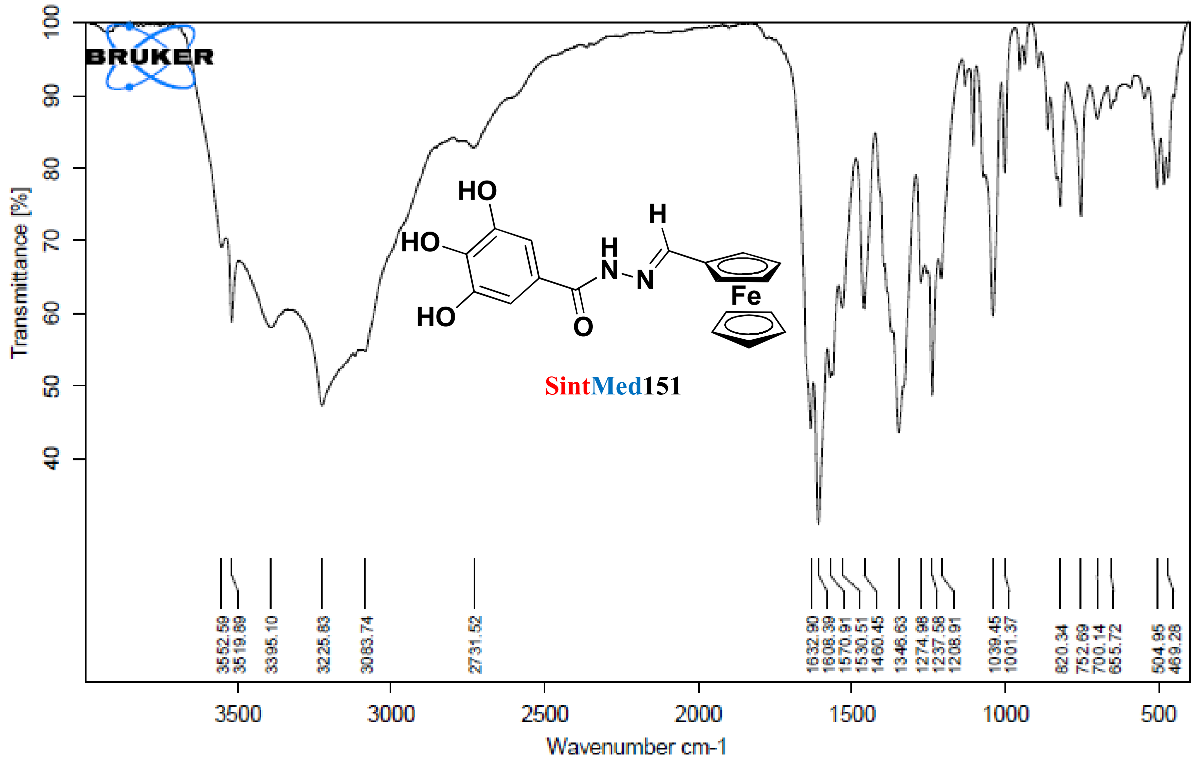

IR spectrum of SintMed151

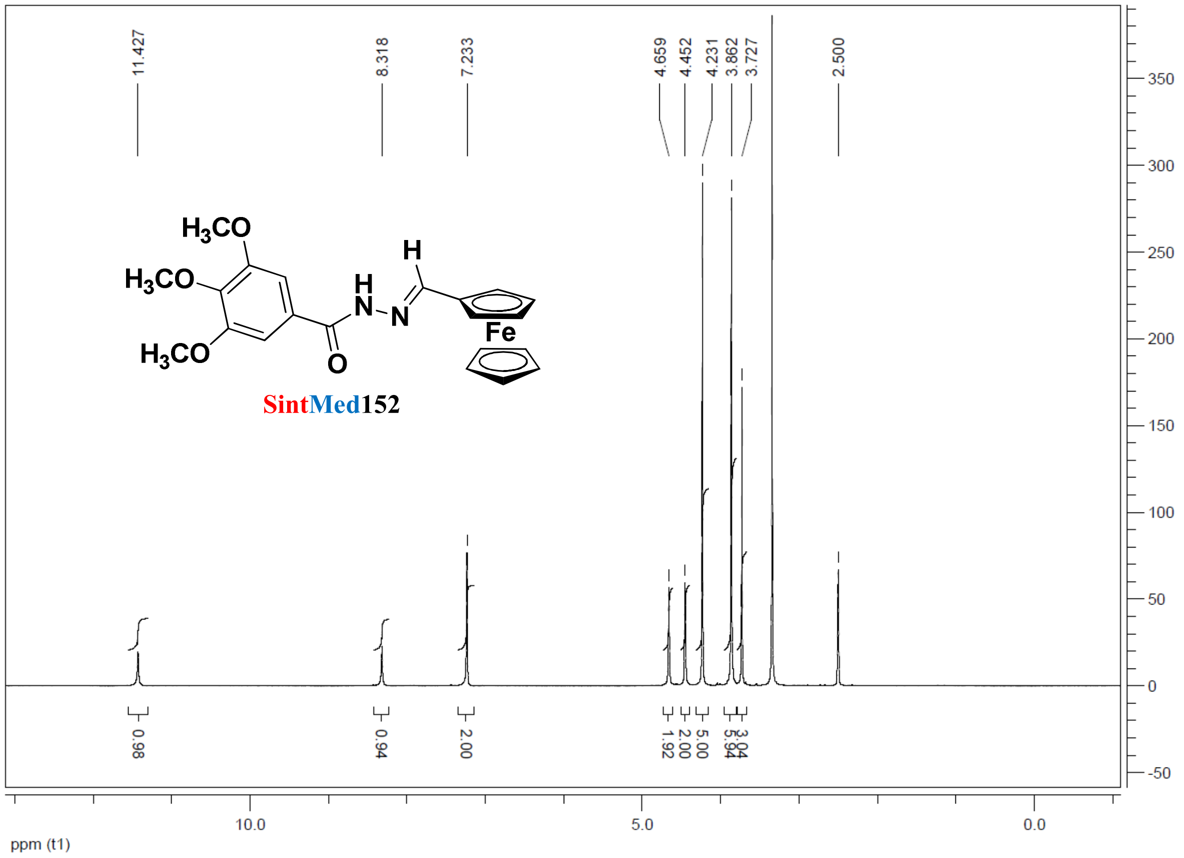

^{1}H NMR spectrum of SintMed152 (400 MHz, DMSO-d_6)

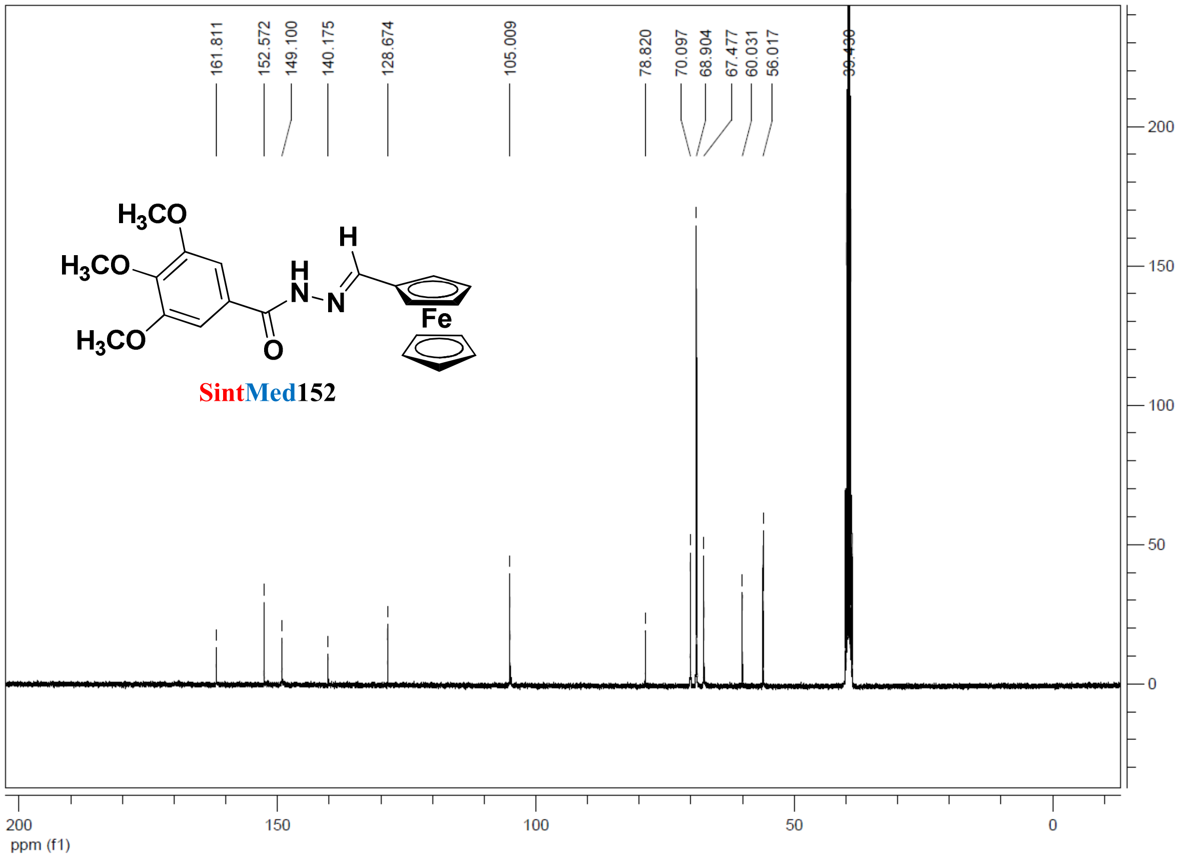

^{13}C NMR spectrum of SintMed152 (100 MHz, DMSO-d_6)

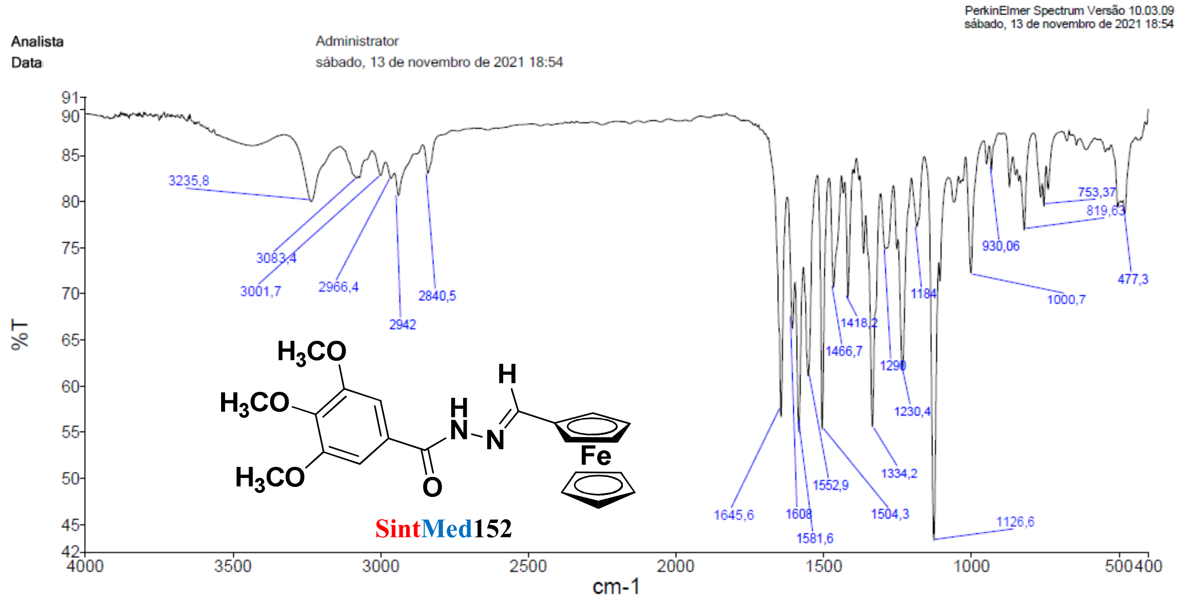

IR spectrum of SintMed152

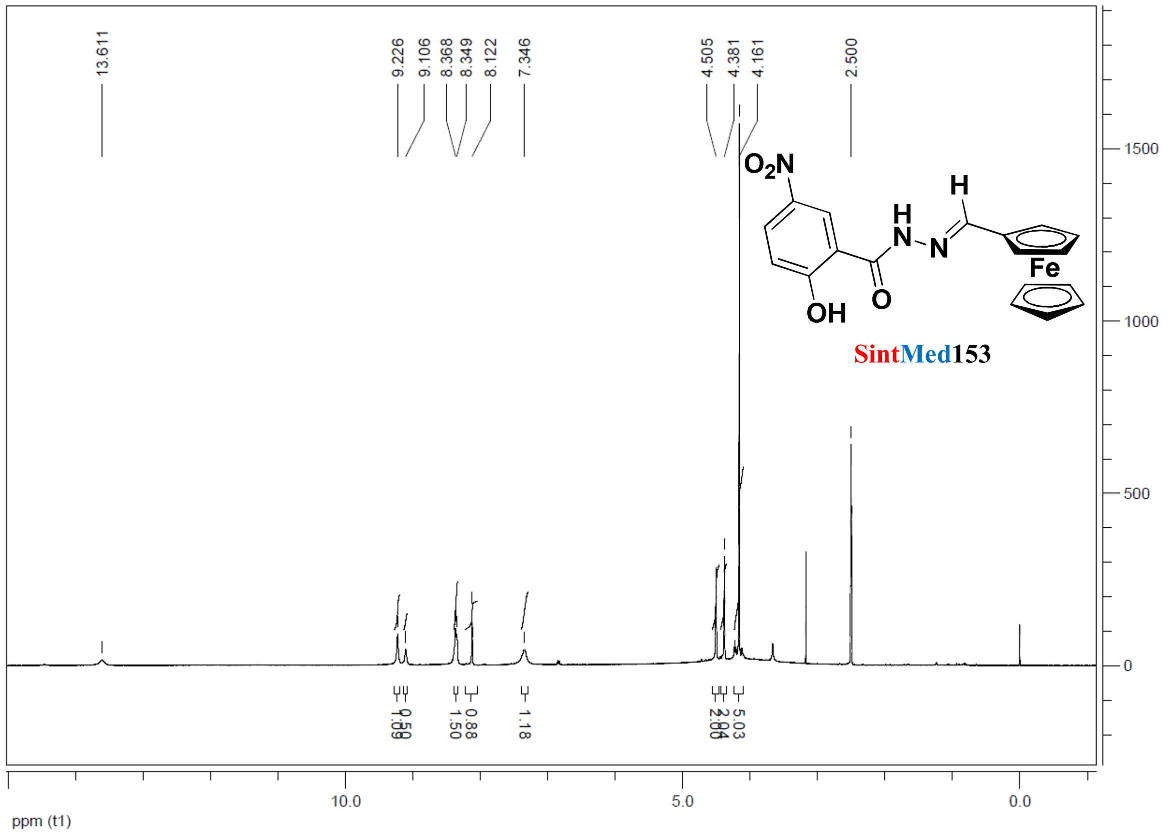

^{1}H NMR spectrum of SintMed153 (400 MHz, DMSO-d_6)

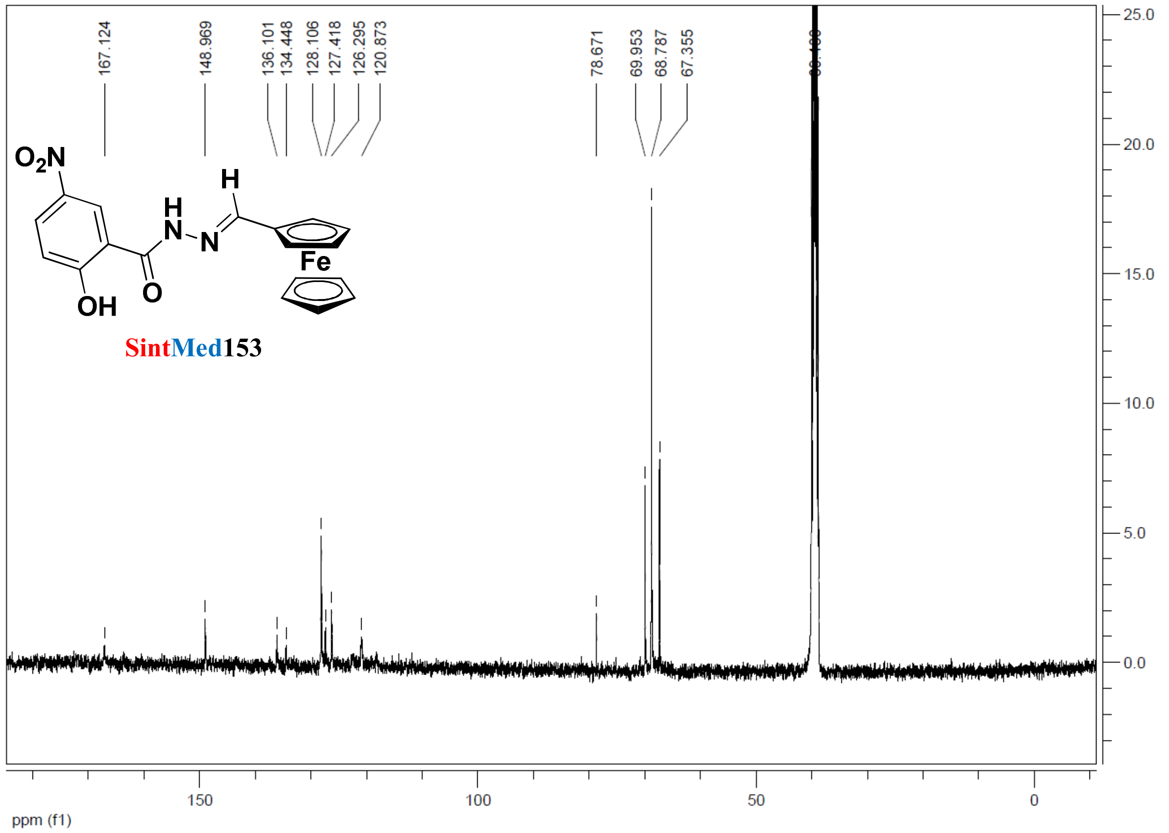

^{13}C NMR spectrum of SintMed153 (100 MHz, DMSO-d_6)

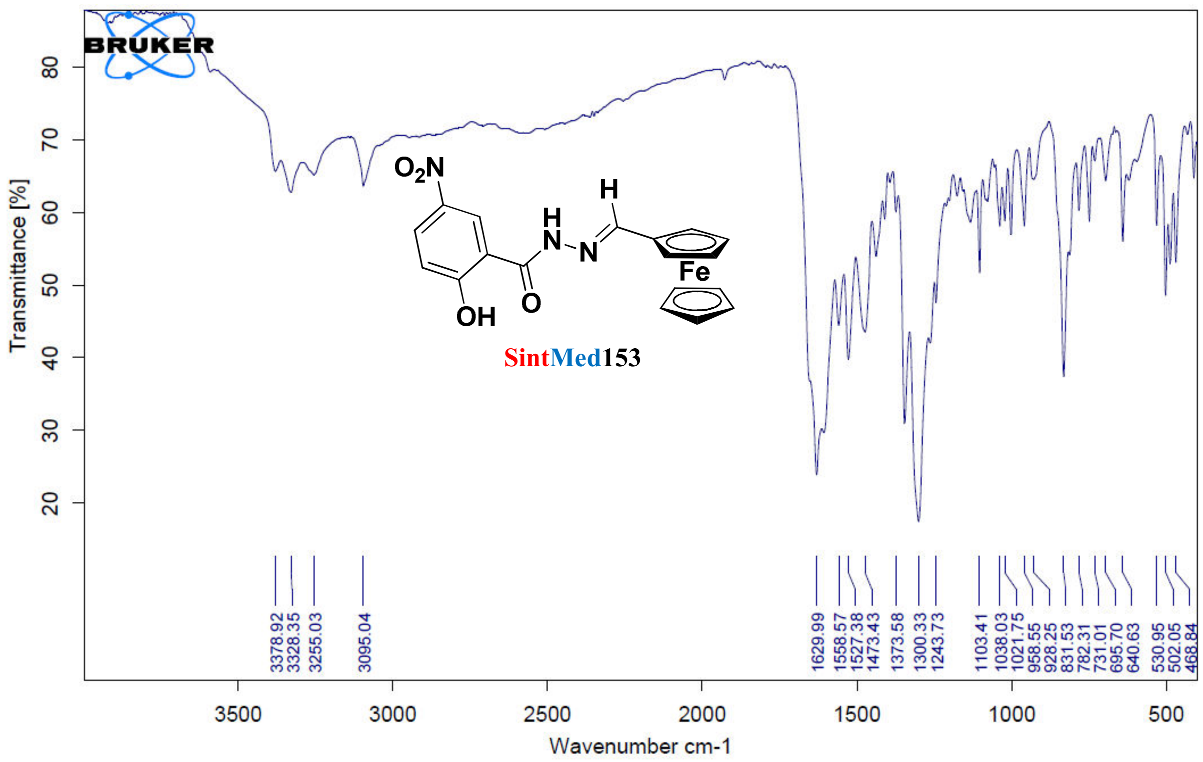

IR spectrum of SintMed153

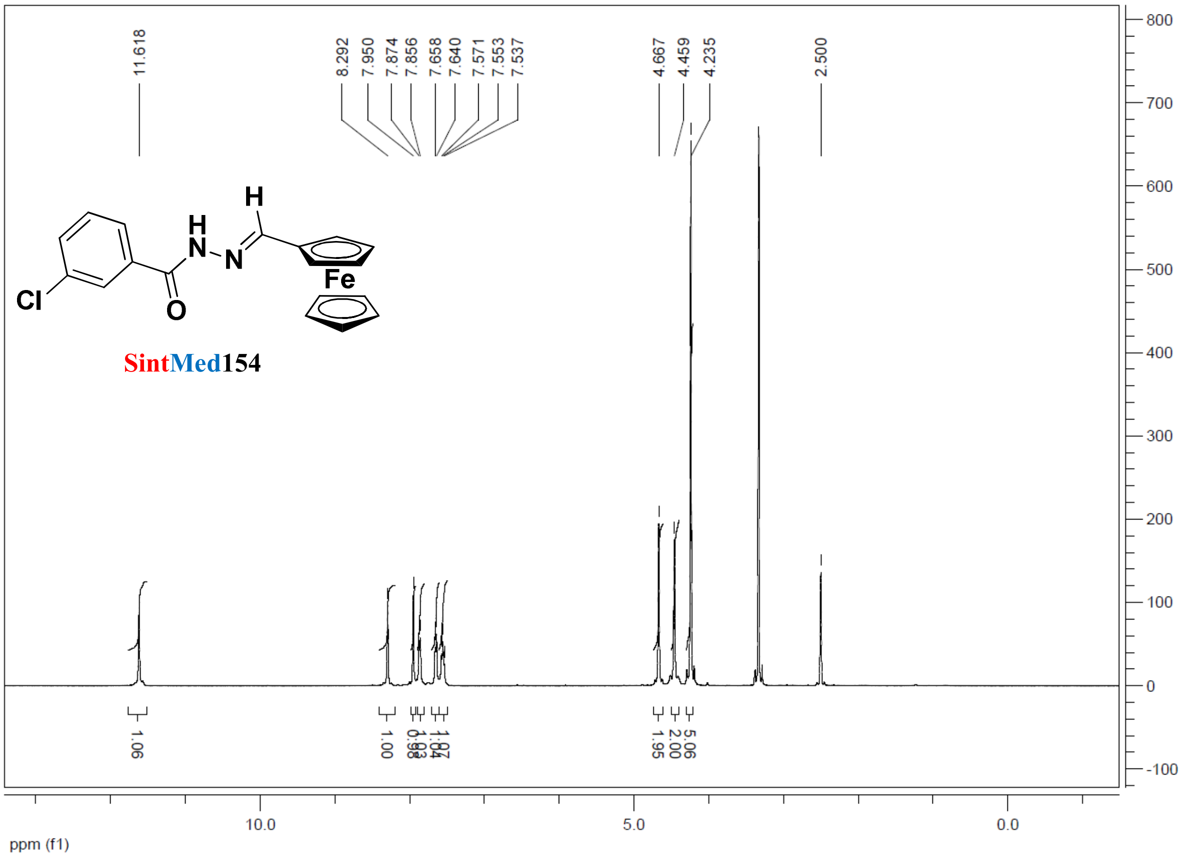

^{1}H NMR spectrum of SintMed154 (400 MHz, DMSO-d_6)

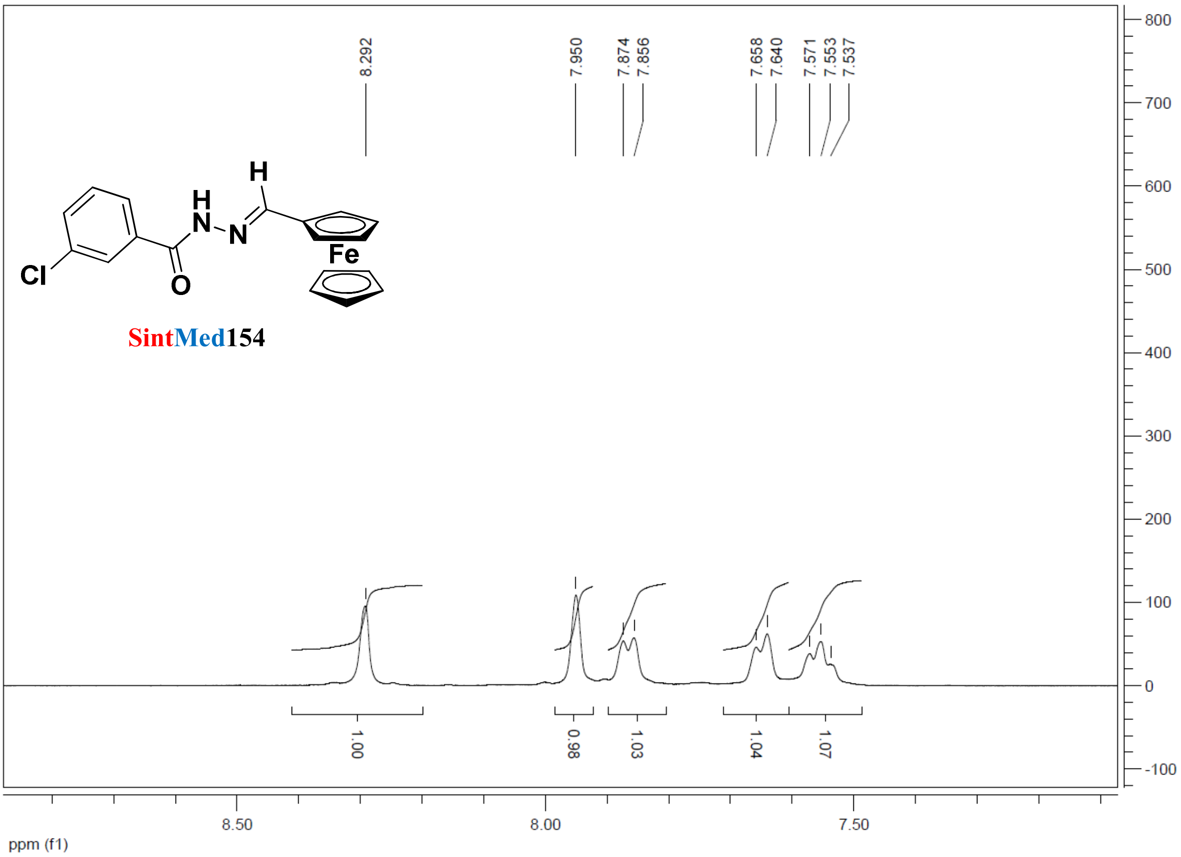

^{1}H NMR spectrum expansion of SintMed154 (400 MHz, DMSO-d_6)

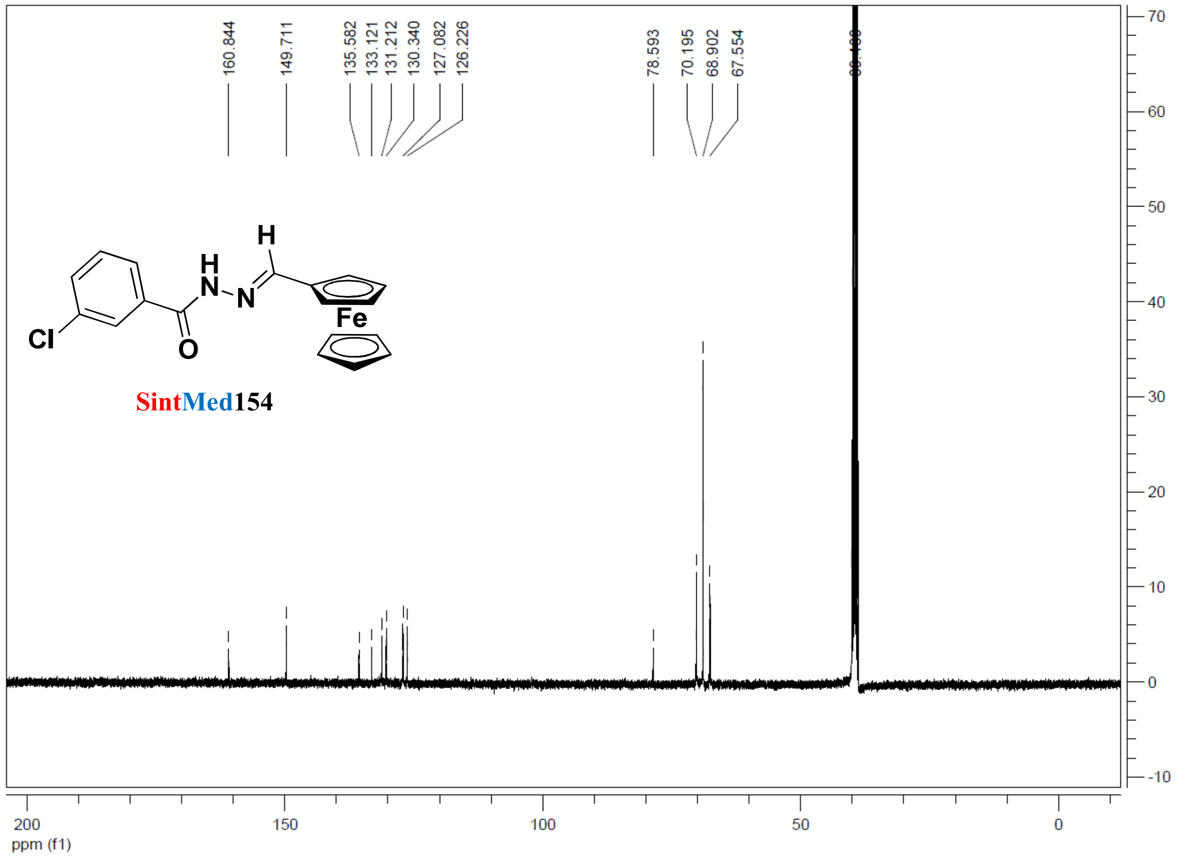

^{13}C NMR spectrum of SintMed154 (100 MHz, DMSO-d_6)

IR spectrum of SintMed154

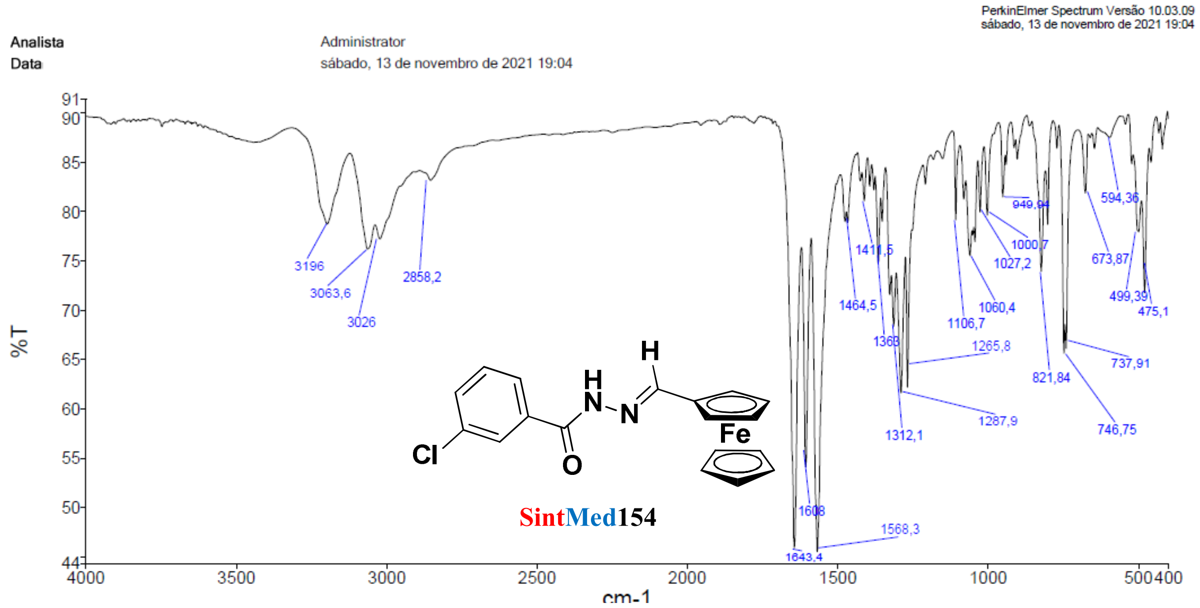

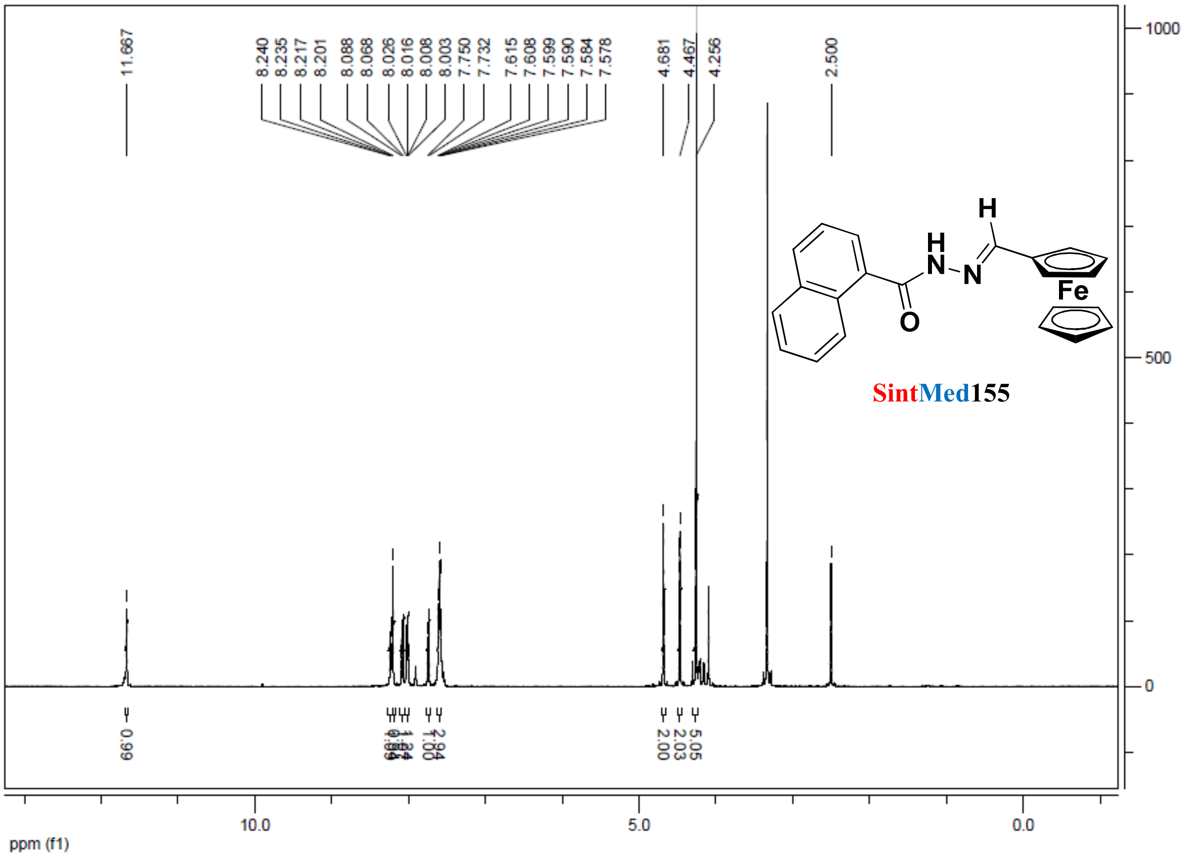

^{1}H NMR spectrum of SintMed155 (400 MHz, DMSO-d_6)

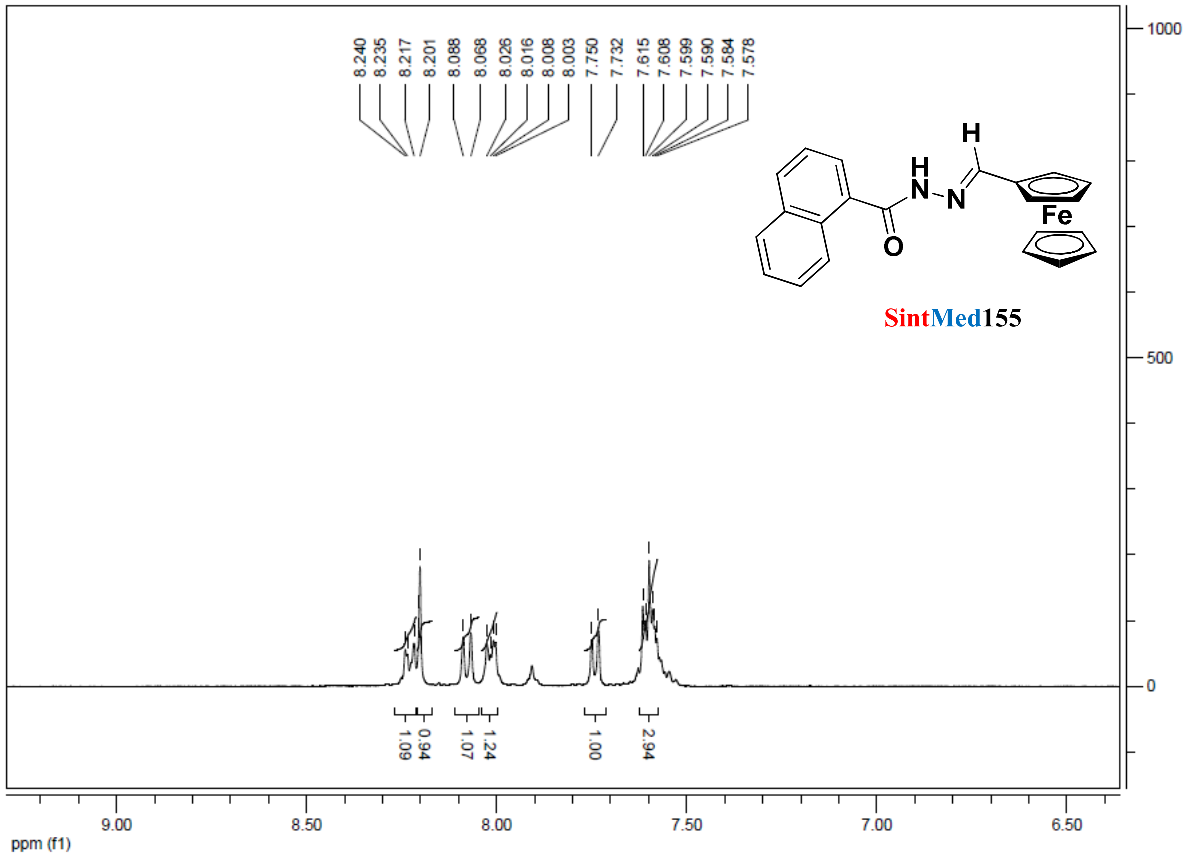

^{1}H NMR spectrum expansion of SintMed155 (400 MHz, DMSO-d_6)

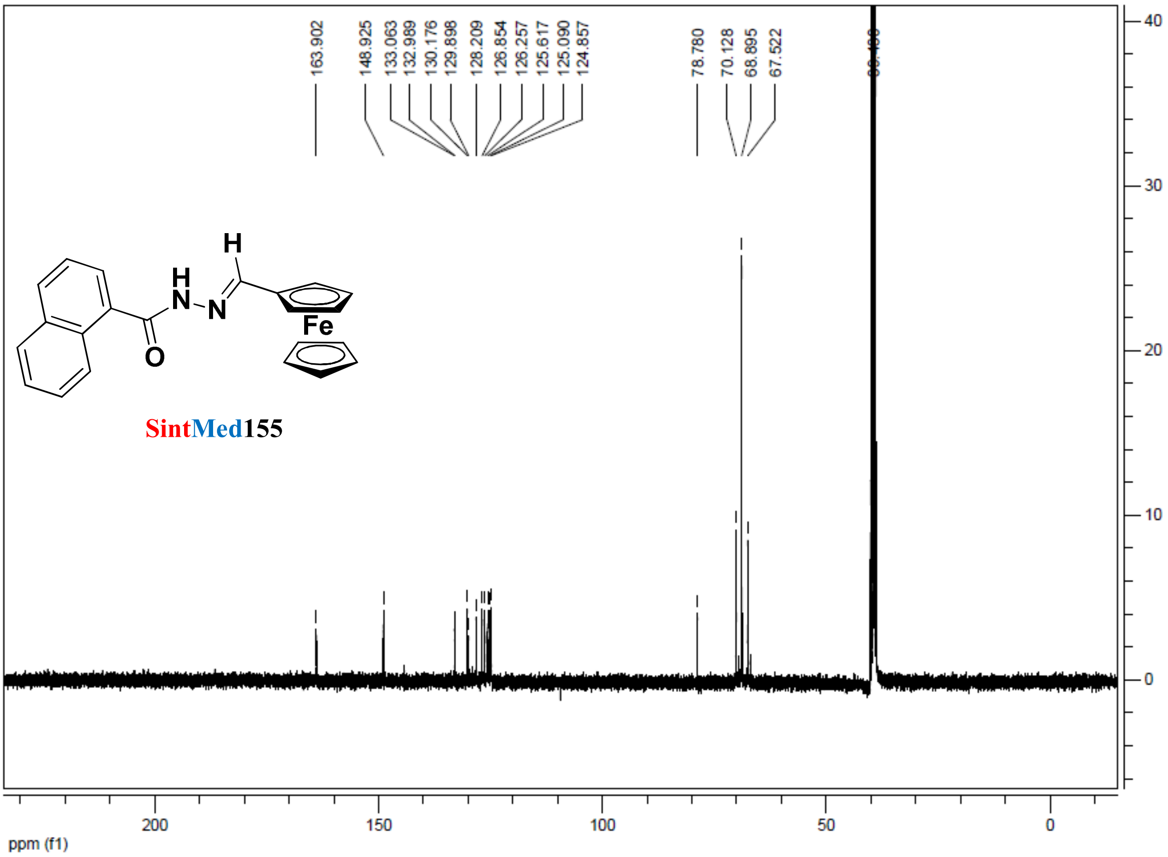

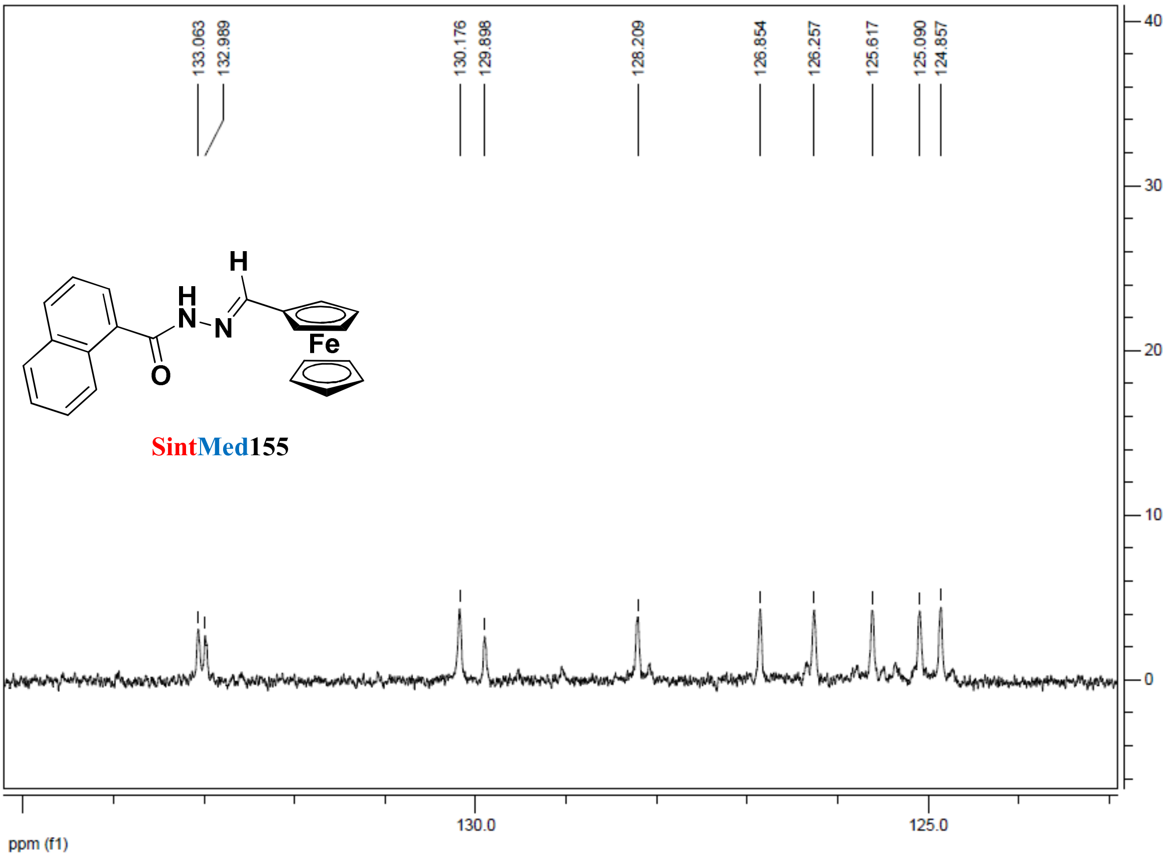

^{13}C NMR spectrum of SintMed155 (100 MHz, DMSO-d_6)

^{13}C NMR spectrum expansion of SintMed155 (100 MHz, DMSO-d_6)

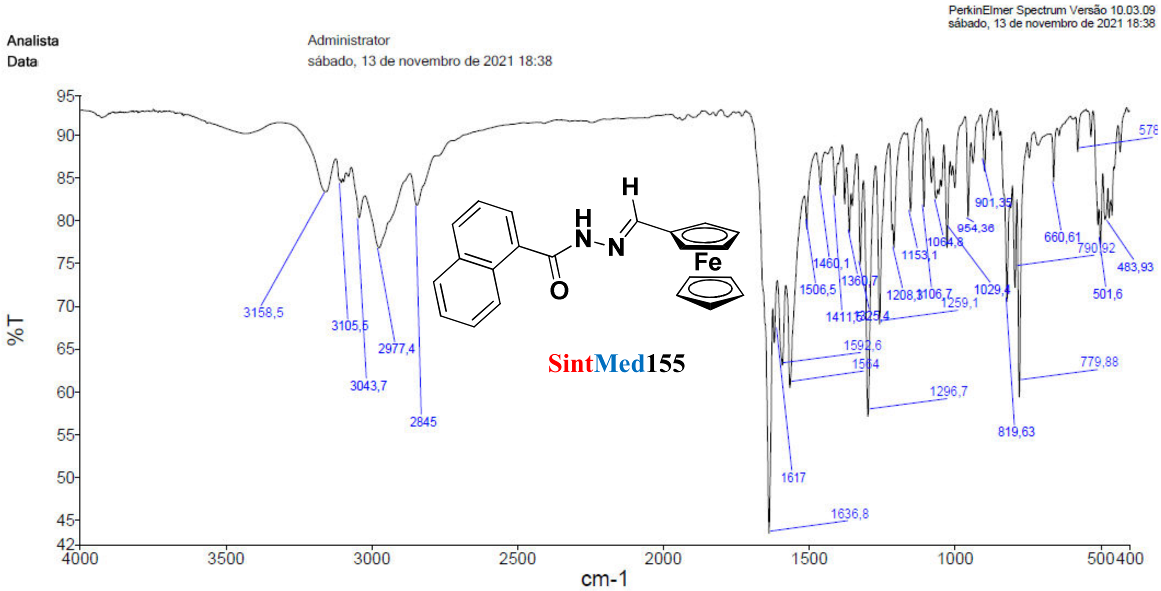

IR spectrum of SintMed155

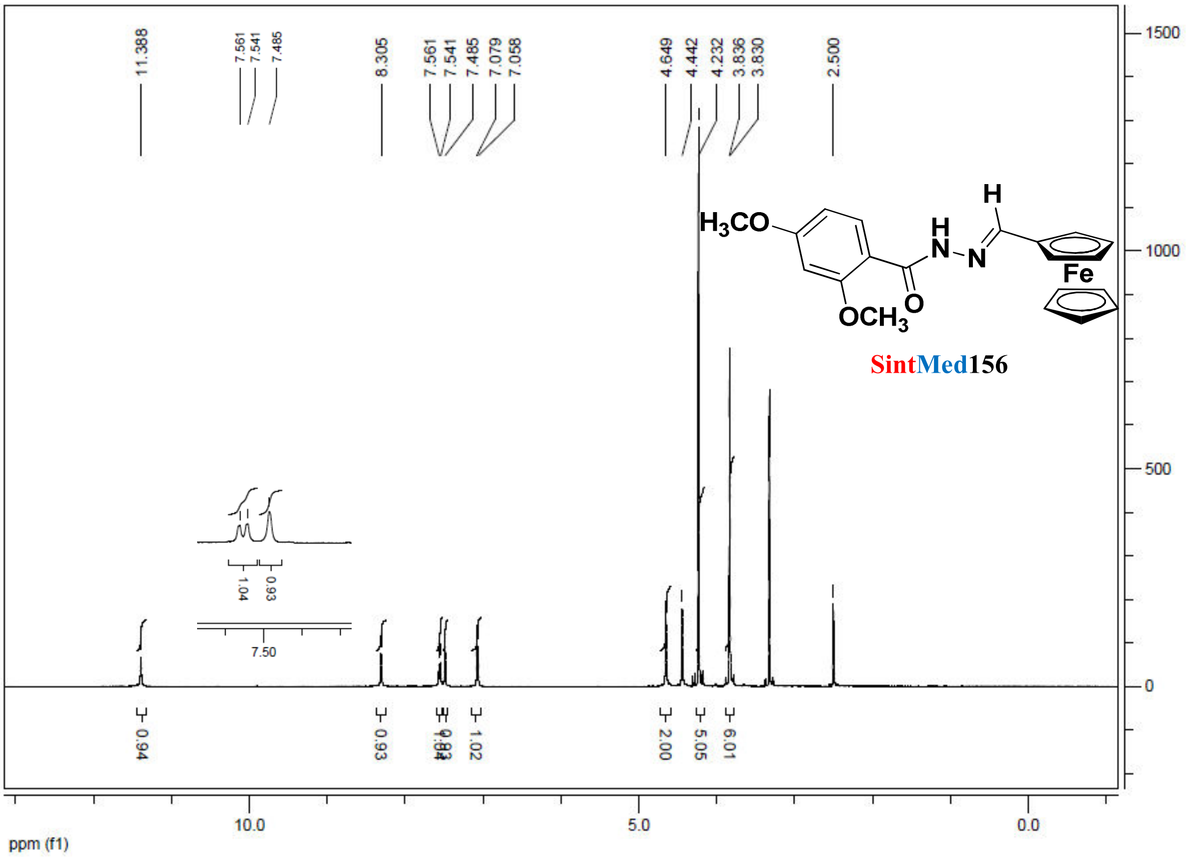

^{1}H NMR spectrum of SintMed156 (400 MHz, DMSO-d_6)

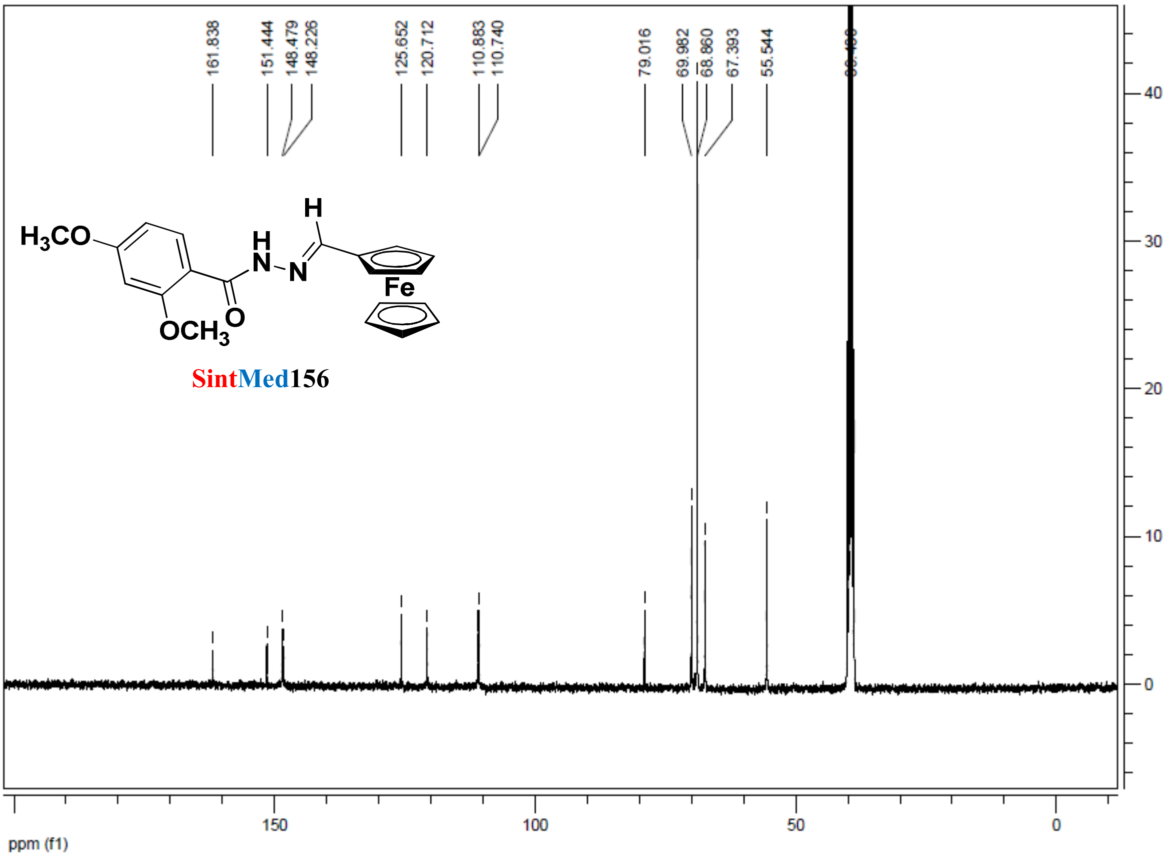

^{13}C NMR spectrum of SintMed156 (100 MHz, DMSO-d_6)

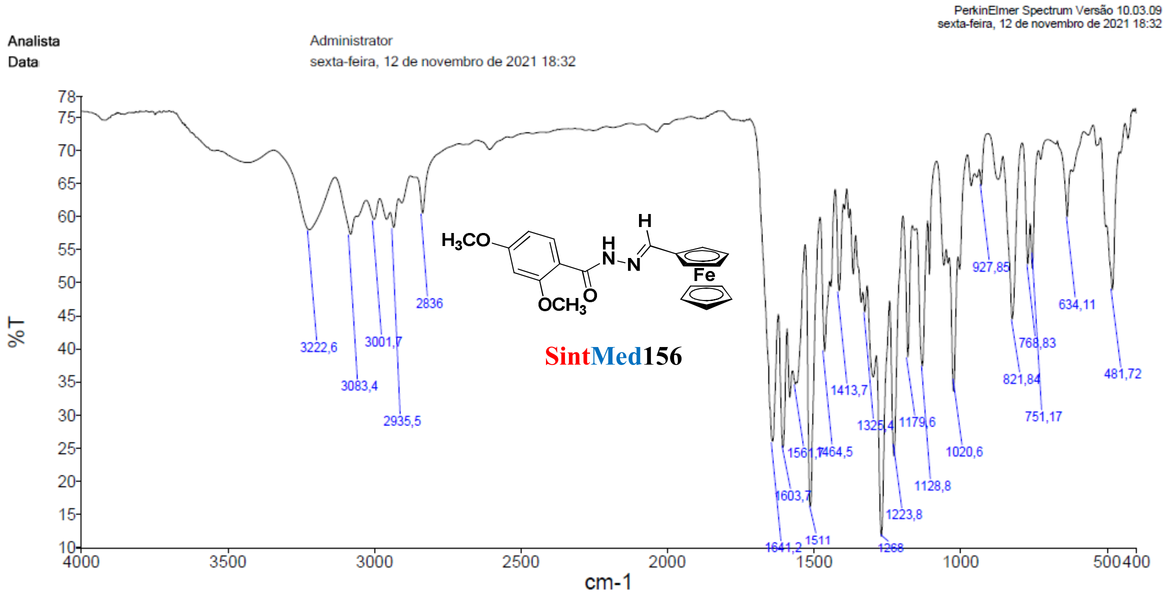

IR spectrum of SintMed156

X-ray crystallographic data of **SintMed149**

The X-ray crystallographic studies reveal that the complex crystallizes in the triclinic system with the P-1 space group and exhibits two molecules in the asymmetric unit. As shown in **Fig. S1**, the obtained crystal structure corroborates with the results from the other characterization techniques. These two molecules of the asymmetric unit are similar, being the main difference in the overlapping of the ferrocene rings, which in one approaches the eclipsed conformation, while the other is more similar to the staggered conformation.

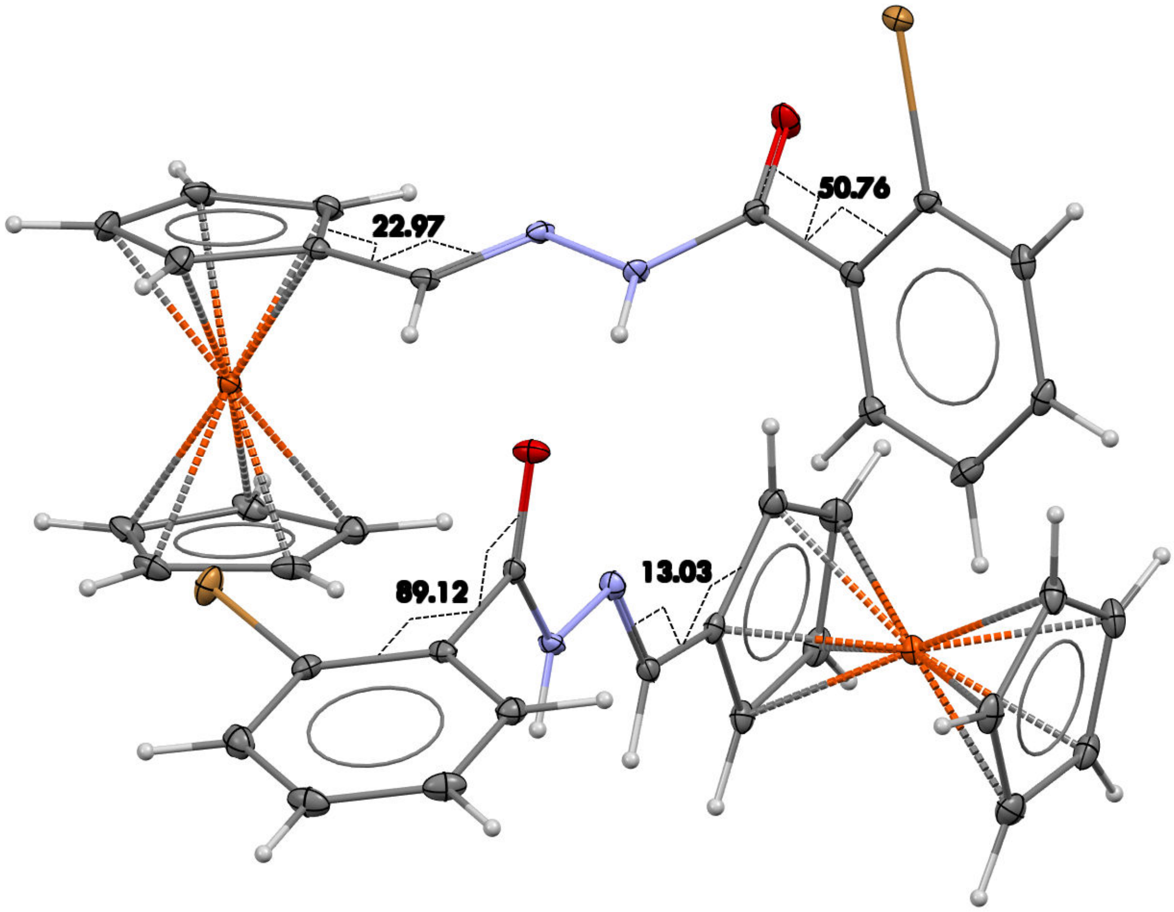

Figure S1. Representation of the asymmetric unit and torsion angles presented by the complex **SintMed149**.

Another important difference between these two molecules is the torsion angles presented between the plane of the ferrocene ring and the C=N bond and the six-member aromatic ring and the C=O bond, as shown in **Figure S1**. These different angles presented by equivalent molecules confirm the high fluxionality of these molecules.

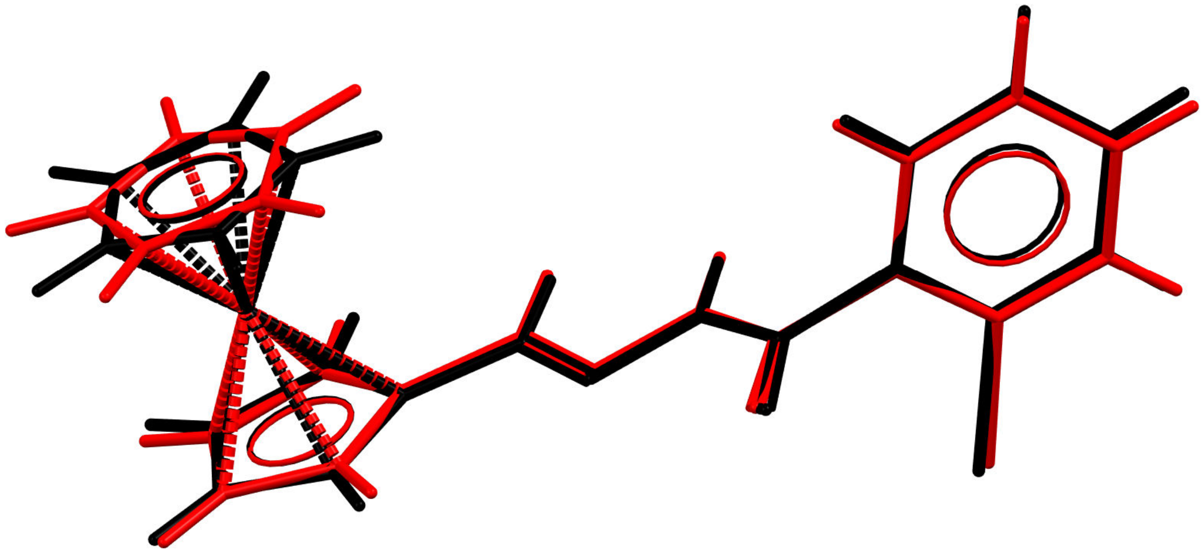

Figure S2. Superposition of the two structures present in the asymmetric unit obtained in the crystallographic studies of compound **SintMed149**.

Table S1. Crystal data and structure refinement for complex **SintMed149.**

CCDC code	2214776
Empirical formula	$C_{18}H_{15}BrFeN_2O$
Formula weight	411.08
Temperature/K	100.01(10)
Crystal system	triclinic
Space group	P-1
a/Å	7.3780(2)
b/Å	10.3437(3)
c/Å	21.0830(6)
α/°	88.551(3)
β/°	89.580(2)
γ/°	81.369(2)
Volume/Å^3	1590.22(8)
Z	4
ρ_{calc}g/cm^3	1.717
μ/mm^{-1}	3.466
F(000)	824.0
Crystal size/mm^3	0.215 × 0.072 × 0.026
Radiation	Mo Kα (λ = 0.71073)
2Θ range for data collection/°	5.484 to 54.2
Index ranges	$-9 \leq h \leq 9$, $-13 \leq k \leq 13$, $-26 \leq l \leq 27$
Reflections collected	28737
Independent reflections	7025 [R_{int} = 0.0389, R_{sigma} = 0.0357]
Data/restraints/parameters	7025/0/415
Goodness-of-fit on F^2	1.028
Final R indexes [I>=2σ (I)]	R_1 = 0.0274, wR_2 = 0.0569
Final R indexes [all data]	R_1 = 0.0363, wR_2 = 0.0591
Largest diff. peak/hole / e Å^{-3}	0.42/-0.31
